# *Listeria monocytogenes* sequence type 1 is predominant in ruminant rhombencephalitis

**DOI:** 10.1038/srep36419

**Published:** 2016-11-16

**Authors:** Margaux Dreyer, Lisandra Aguilar-Bultet, Sebastian Rupp, Claudia Guldimann, Roger Stephan, Alexandra Schock, Arthur Otter, Gertraud Schüpbach, Sylvain Brisse, Marc Lecuit, Joachim Frey, Anna Oevermann

**Affiliations:** 1 Division of Neurological Sciences, Department of Clinical Research and Veterinary Public Health, Vetsuisse Faculty, University of Bern, Switzerland; 2 Graduate School for Cellular and Biomedical Sciences, Theodor Kocher Institute, University of Bern, Switzerland; 3 Institute of Veterinary Bacteriology, Department of Infectious Diseases and Pathobiology, University of Bern, Switzerland; 4 Institute for Food Safety and Hygiene, University of Zurich, Switzerland; 5 Department of Pathology, AHVLA Lasswade, Pentlands Science Park, Bush Loan, Penicuik Midlothian, United Kingdom; 6 AHVLA Shrewsbury Kendal Road, Harlscott, Shrewsbury, Shropshire, United Kingdom; 7 Veterinary Public Health Institute, University of Bern, Switzerland; 8 Institut Pasteur, Microbial Evolutionary Genomics, Paris, France; 9 Institut Pasteur, Biology of Infection Unit, Paris, France; 10 Inserm U1117, Paris, France; 11 French National Reference Center and WHO Collaborating Center Listeria, Institut Pasteur, Paris, France; 12 Paris Descartes University, Sorbonne Paris Cité, Division of Infectious Diseases and Tropical Medicine, Necker-Enfants Malades University Hospital, Institut Imagine, Paris, France

## Abstract

*Listeria (L.*) *monocytogenes* is an opportunistic pathogen causing life-threatening infections in diverse mammalian species including humans and ruminants. As little is known on the link between strains and clinicopathological phenotypes, we studied potential strain-associated virulence and organ tropism in *L. monocytogenes* isolates from well-defined ruminant cases of clinical infections and the farm environment. The phylogeny of isolates and their virulence-associated genes were analyzed by multilocus sequence typing (MLST) and sequence analysis of virulence-associated genes. Additionally, a panel of representative isolates was subjected to *in vitro* infection assays. Our data suggest the environmental exposure of ruminants to a broad range of strains and yet the strong association of sequence type (ST) 1 from clonal complex (CC) 1 with rhombencephalitis, suggesting increased neurotropism of ST1 in ruminants, which is possibly related to its hypervirulence. This study emphasizes the importance of considering clonal background of *L. monocytogenes* isolates in surveillance, epidemiological investigation and disease control.

*Listeria (L.*) *monocytogenes* is an opportunistic pathogen that may cause life-threatening infections in many mammalian species upon ingestion^1^,^2^. Listeriosis is of major importance in humans and in farmed ruminants^3^, in which it is associated with gastroenteritis, abortions, bacteremia, mastitis and central nervous system (CNS) infections (neurolisteriosis)^4^,^5^,^6^,^7^. In humans, *L. monocytogenes* has the highest hospitalization and mortality rates amongst food-borne pathogens^8^,^9^. Surveillance and control prove to be challenging due to the environmental lifestyle of *L. monocytogenes*, which colonizes diverse environments including soil, water, food processing plants, mammalian intestinal tracts and faeces^8^,^10^,^11^,^12^. In consequence, *L. monocytogenes* is frequently found as a contaminant of human food and animal feed.

*L. monocytogenes* is divided into 13 serotypes^13^ and four phylogenetic lineages^14^,^15^,^16^,^17^. Regulations consider all *L. monocytogenes* strains an equally serious threat for public health^18^, although an increasing number of studies indicate differences in ecology and virulence between *L. monocytogenes* strains^7^,^16^,^19^,^20^,^21^,^22^. Most lineage I strains are overrepresented in human clinical infections and in ruminant neurolisteriosis, while a majority of lineage II strains are associated with food contaminations and the environment^7^,^14^,^23^,^24^. The minor lineages III and IV are rarely isolated and have been linked to animals^14^. Multilocus sequence typing (MLST), which categorizes isolates into sequence types (STs) and clonal complexes (CCs, hereafter equated to clones) based on sequence data of seven housekeeping genes^25^,^26^,^27^, has shown that *L. monocytogenes* has a clonal population structure with hypervirulent and hypovirulent clones and that the distribution of certain clones significantly varies between human clinical infection and food^7^,^25^,^26^. However, little is known about strain-associated clinicopathological phenotypes in *L. monocytogenes*.

To identify clones with higher virulence or organ tropism, we comparatively studied *L. monocytogenes* isolates from naturally occurring ruminant infections and from their farm environment. The most frequent clinicopathological phenotypes of listeriosis in ruminants are abortions and neurolisteriosis^3^,^8^. The latter is one of the most prevalent and fatal CNS infections in ruminants and is typically characterized by encephalitis of the brainstem (rhombencephalitis)^28^,^29^,^30^,^31^. Other manifestations such as gastroenteritis, bacteremia and mastitis are only sporadically encountered in these species^32^,^33^,^34^,^35^. Additionally, clinically healthy ruminants may carry *L. monocytogenes* in their gastrointestinal tract and shed them into the environment^36^,^37^,^38^.

We compared *L. monocytogenes* isolates from well-defined rhombencephalitis cases and non-encephalitic cases to isolates from healthy ruminants and from the direct ruminant environment in order to identify STs associated with disease or specific tropisms. Additionally, we screened genes that have been implicated in *L. monocytogenes* virulence^39^ for polymorphisms and recombination that may explain variations in virulence and organ tropism between strains. Results show that ruminants are exposed to a broad range of genetically diverse strains in the farm environment, yet a particular genotype, ST1, is predominant in rhombencephalitis cases, but not in other disease manifestations. These data suggest increased neurotropism of ST1 strains in ruminants, which might be related to its hypervirulence.

## Results

### Differential distribution of STs between diseased ruminants and environment

**Figure 1:**
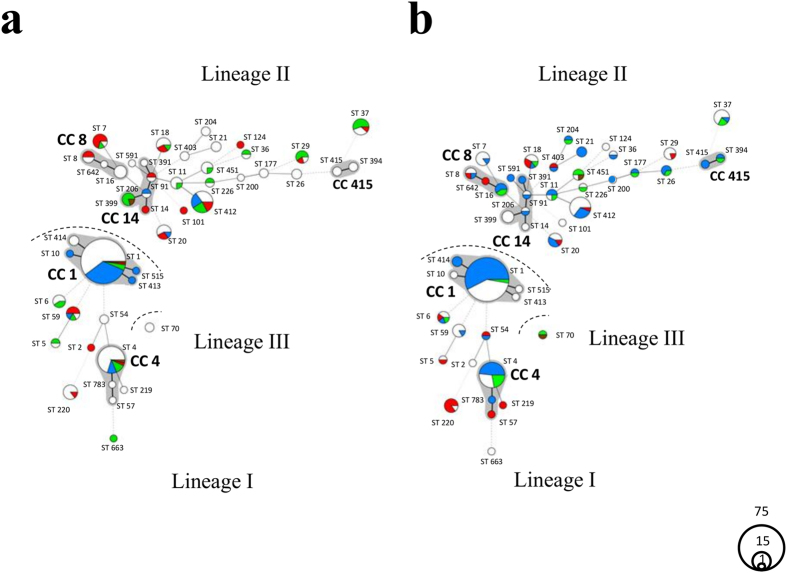
Minimum spanning tree (MST) of 248 *L. monocytogenes* isolates based on multilocus sequence typing (MLST) analysis. Circles represent sequence types (STs) and their size corresponds to the number of isolates present in each ST. The lines between different STs represent phylogenetic relationships, bold lines indicate one mismatch in the seven housekeeping genes, plain lines two mismatches, discontinuous lines three mismatches and light discontinuous lines four or more mismatches. Grey zones surrounding multiple STs, represent clonal complexes (CCs), which contain STs with a single mismatch in the seven loci. The three evolutionary lineages are indicated. Isolates of ruminant rhombencephalitis cases are represented in blue (n = 140), non-encephalitic ruminant clinical cases in red (n = 47), ruminant faecal isolates in brown (n = 5) and isolates of the ruminant farm environment in green (n = 56). (**a**) MST of cattle isolates. Isolates of rhombencephalitis cases are represented in blue (n = 39), non-encephalitic cattle clinical cases in red (n = 28), cattle faecal isolates in brown (n = 3), isolates of their environment in green (n = 33) and small ruminant-associated isolates in white (n = 145). (**b**) MST of small ruminant isolates. Isolates of rhombencephalitis cases are represented in blue (n = 101), non-encephalitic clinical cases in red (n = 19), faecal isolates in brown (n = 2), isolates of their environment in green (n = 23) and cattle-associated isolates in white (n = 103).

MLST was performed of 187 ruminant clinical isolates from cases with various clinicopathological outcomes (rhombencephalitis, abortions, gastroenteritis, and mastitis), five faecal isolates of clinically healthy ruminants and 56 environmental isolates from ruminant farms (Text. S1, Table S1). Eight to 20 alleles per locus were identified resulting in 45 STs. These STs were distributed over five CCs and 29 singletons within three lineages (lineage I n = 141, lineage II n = 105, lineage III n = 2; Fig. 1). Lineage I showed an increased clustering compared to lineage II, which contained a higher number of distantly related STs. The prevalence varied considerably between STs as 51% (n = 126) of all isolates belonged to only three STs (ST1: 33%, n = 81; ST4: 11%, n = 28; ST412: 7%, n = 17) while the remaining 49% of isolates were distributed over 42 STs (Table S1). The clustering of isolates in these three STs (ST1, ST4, ST412) was particularly striking in clinical isolates (84%) compared to environmental isolates (16%).

**Figure 2:**
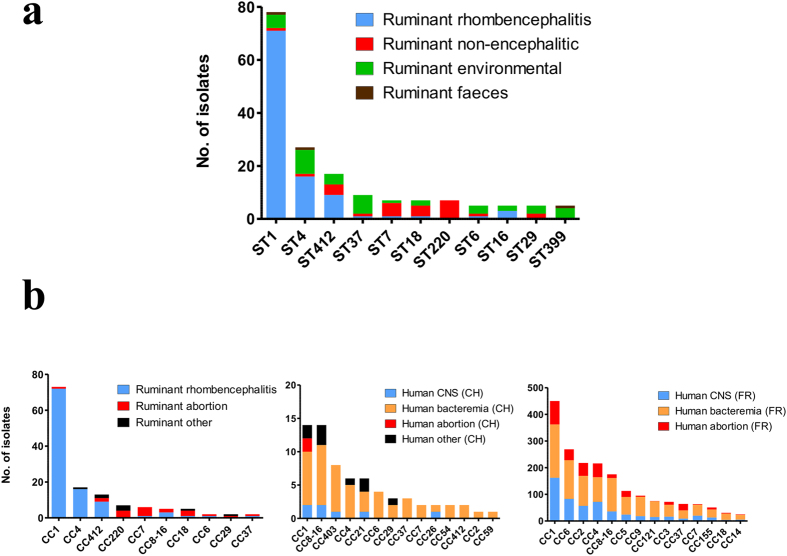
Frequency of *L. monocytogenes* isolates according to their source of isolation in the most prevalent sequence types (STs) and clonal complexes (CCs). (**a**) Non-homogeneous distribution of STs in ruminant-associated isolates. The 11 most prevalent STs are arranged according to their abundance. Blue = ruminant rhombencephalitis (n = 134), red = ruminant non-encephalitic infections (n = 27), green = ruminant-associated environment (n = 40), brown = faeces (n = 4). (**b**) Divergent distribution of CCs in ruminant (left) and human clinical isolates from Switzerland (CH, middle)^40^ and France (FR, right)^7^. Blue = ruminant rhombencephalitis/human central nervous system (CNS) isolates, red = abortions, black = other infection sources, orange = human bacteremia.

Clinical and environmental isolates partially overlapped in their ST distribution, with 17 STs (38%) containing isolates from both sources. However, the relative prevalence of STs varied strongly between clinical and environmental sources and, notably, between rhombencephalitic and non-encephalitic outcomes of ruminant listeriosis (Figs 1 and 2a). The genetic diversity of isolates was significantly smaller in rhombencephalitis cases (71%; 95% CI ranging from 70.5% to 71.4%) than in non-encephalitic cases (96%; 95% CI ranging from 94.8% to 96.1%) and environmental sources (93%; 95% CI ranging from 90.1% to 95.6%). Rhombencephalitis isolates were significantly overrepresented in lineage I compared to lineage II and, particularly, in ST1 (CC1) of lineage I, which contained 53% (n = 74) of all rhombencephalitis isolates (Figs 1 and 2a, Table 1). Strikingly, 91% of ST1 were of rhombencephalitis origin, while only 36% of the other STs were of rhombencephalitis origin (*p-*value < 0.001). Relative ST distribution in rhombencephalitis differed between hosts (Fig. 1): while in cattle 84% of ST1, but only 16% of other STs were of rhombencephalitis origin (*p-*value < 0.001), the variety of other STs contributing to rhombencephalitis was higher in small ruminants. Yet, ST1 was predominant in rhombencephalitis also in these species. Here, 96% of ST1 were of rhombencephalitis origin, while the proportion of rhombencephalitis isolates in other STs was lower (56%, *p-*value < 0.001). Compared to most other STs, ST1 contained strikingly few isolates from non-encephalitic clinical sources (1.3%), environment (6%) and faeces (1.3%) (Figs 1 and 2a). Two further STs, in which rhombencephalitis isolates clearly prevailed, were ST4 from CC4 (61%) and ST412 from CC412 (53%). However, as they only accounted for 12% (ST4) and 7% (ST412) of isolates, association of rhombencephalitis with these STs was not statistically significant. ST412 was a particular lineage II strain, as in contrast to other lineage II STs it was mainly isolated from rhombencephalitis. ST4, ST412 and rarely ST1 were also isolated from the environment (Fig. 2a). Interestingly, in contrast to ST412, environmental and faecal isolates belonging to ST1 and ST4 were isolated in farms with ongoing^35^ or recent outbreaks, suggesting recent contamination from clinical cases.

In contrast to the rhombencephalitic isolates, isolates from non-encephalitic origin, environment and faeces were significantly associated with lineage II (Table 1). Some STs contained mainly (STs 7, 18) or only (ST220) clinical isolates from non-encephalitic origin. ST220 was significantly associated with non-encephalitic pathologies when compared to all other STs (*p-*value < 0.001), contrasting with the association of ST1 with rhombencephalitis. STs 399 and 451 were associated only with environmental and faecal isolates, and 78% of ST37 isolates originated from the ruminant environment and faeces. The distribution of these three STs between clinical and environmental/faecal isolates was significantly different (ST37: *p-*value < 0.001; ST399: *p-*value < 0.01; ST451: *p-*value < 0.01) when compared to the remaining STs.

Comparison with multilocus variable number of tandem repeat (MLVA) revealed that (with very few exceptions) lineage II corresponded to MLVA complex C, whereas CC1 and CC4 corresponded to MLVA complexes A and B, respectively^23^ (Fig. S1). Consistent with the literature, serotypes 1/2b, 3b, 4b, 4d, 4e and 7 corresponded to lineage I, whereas serotypes 1/2a, 1/2c, 3a, and 3c corresponded to lineage II. Within lineage I, most isolates belonged to serotype group 4b, 4d, 4e and within lineage II, most isolates belonged to serotype group 1/2a and 3a (Fig. S1a, Table S1).

### Differential distribution of STs in human-associated and ruminant-associated isolates

When compared with Swiss and French human isolates from previous studies^7^,^40^, there was some overlap of CCs between human and ruminant clinical isolates, yet the distribution differed (Fig. 2b). Due to the high prevalence of CC1 and CC4 the genetic diversity of ruminant clinical isolates (81%; 95% CI ranging from 80.8% to 81.1%) was significantly lower than the genetic diversity of human clinical isolates (92%; 95% CI ranging from 90.7% to 92.1%). Although CC1 was also amongst the predominant CCs in human clinical isolates (CH: 15%, FR: 20%) (Fig. 2b), its prevalence was significantly higher in ruminant infections (32%, *p*-value < 0.001). The relative dominance of CC1 in ruminants versus humans was strikingly clear in isolates from neurolisteriosis (Fig. 2b). Additionally, CC220 and CC412 (*p*-value < 0.001), which were amongst the most common CCs in ruminant clinical infection, were significantly less prevalent in human infection. In contrast, CCs 2, 3, 5, 6, 7, 9, 29, 121 and 155 were significantly associated with human infection when compared to animal infections (*p*-values < 0.05). Some of these CCs were observed in ruminants, but most were rare or absent amongst the ruminant clinical isolates.

### Isolates of rhombencephalitis-associated STs 1, 4 and 412 are hyperinvasive and hyper-replicative in a bovine macrophage cell line compared to isolates from environment-associated STs 18 and 37

**Figure 3:**
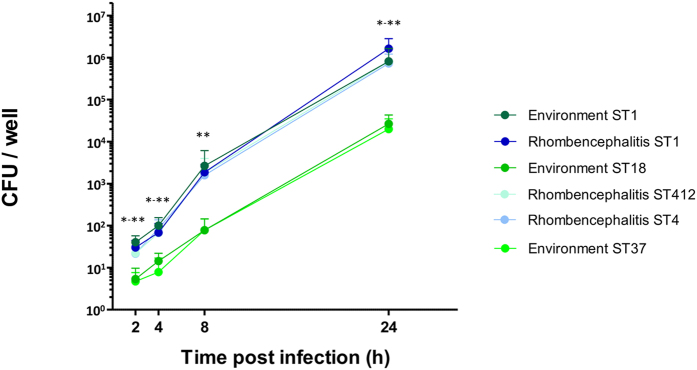
CFUs per well of six isolates in the bovine macrophage (BoMac) cell line. CFUs were enumerated from cell lysates at indicated time points post infection, three independent experiments were performed in triplicates. Error bars indicate 95% SEM. *p-value < 0.05 (**2 h**: ST1 rhombencephalitis VS. ST18 and ST37; **4 h**: ST1 environment and ST4 Vs. ST18, ST1 rhombencephalitis and ST412 Vs. ST37; **24 h**: ST1 Vs. ST18, ST4 Vs. ST37), **p-value < 0.01 (**2 h**: ST1 environment VS. ST18 and ST37; **4 h**: ST1 environment and ST4 Vs. ST37; **8 h**: ST1 and ST4 Vs. ST18 and ST37; **24 h**: ST1 Vs. ST37).

To test whether the association of STs with rhombencephalitis could be attributed to higher invasiveness and increased intracellular replication, we selected six representative cattle isolates based on their ST and source of isolation (rhombencephalitis ST1, ST4 and ST412, environment ST1, ST18 and ST37) and analysed them in a gentamicin protection assay using the bovine macrophage (BoMac) cell line. All six isolates infected BoMac cells. However, invasion and infection kinetics were different between STs (Fig. 3). Significantly more CFUs were recovered from BoMac cells at 2 h when infected with isolates from rhombencephalitis-associated STs 1, 4 and 412, regardless of the source of isolation (rhombencephalitis or environment), indicating that they are more efficient in invasion than isolates from environment-associated STs (ST18, ST37). Intracellular replication between 2 and 24 h post infection was stronger in the ST1, 4 and 412 isolates, which showed a 20 000 to 54 000 (ST1environment/rhombencephalitis) fold increase in CFU numbers over the 22 h incubation period compared to a 4 000 to 5000 fold increase in the ST37 and ST 18 isolates. The difference in recovered CFU numbers between the rhombencephalitis-associated STs and the environment-associated STs increased to up to two orders of magnitude at 24 h post infection. Differences in CFU counts were significant at all time points when ST1, ST4 and ST412 were compared to ST18 and 37 (Fig. 3).

### Virulence-associated gene variations are associated with ST

**Figure 4:**
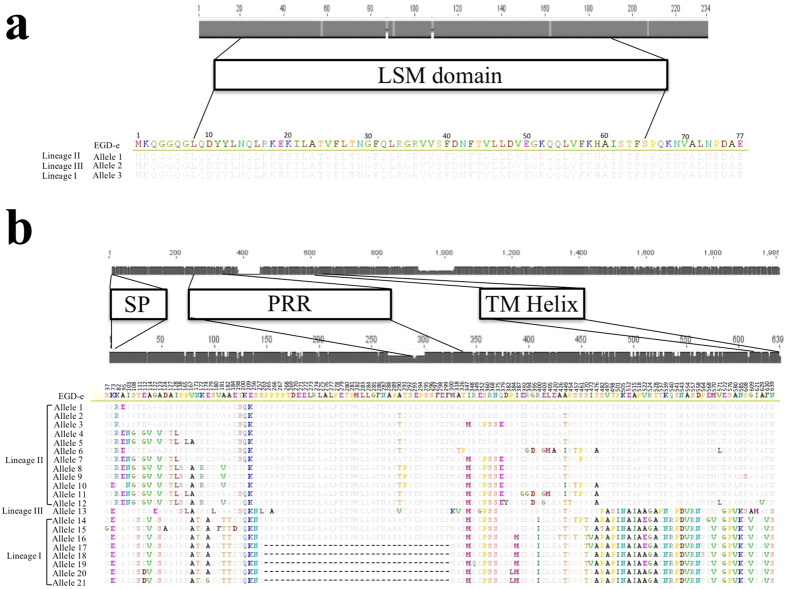
Distribution of polymorphisms in the *hfq* and *actA* genes (grey bars). The scale above the grey bars indicates nucleotide numbers. Below the functional domains, predicted amino acid sequences of the *hfq* and *actA* alleles are aligned to the reference strain EGD-e, and scales indicate amino acid number. (**a**) Polymorphisms within the 234 nucleotides of *hfq* are indicated as vertical bars in the grey bar. The functional domain LSM (like-Sm) is represented by a white box. While on the nucleotide level every lineage has a specific allele, none of the polymorphisms results in amino-acid changes and the amino-acid sequence is conserved across lineages. (**b**) Synonymous and non-synonymous nucleotide polymorphisms across the 26 *actA* alleles are shown as vertical bars in the upper grey bar. The functional domains (signal peptide, SP; proline rich repeats, PRR and transmembrane domain, TM) are shown in white boxes. The scale above the lower grey bar indicates amino-acid numbers and vertical bars correspond to non-synonymous nucleotide polymorphisms. Below, the amino-acid sequences of the *actA* 21 alleles are aligned to the reference strain EGD-e. Amino acid polymorphisms (or non-synonymous mutations) are represented in colour and gaps by “-”.

Using a subset of 94 isolates from 25 STs (Table S1) with available next generation sequencing (NGS) data, we analyzed 45 *L. monocytogenes* virulence-associated genes^39^ (Table S2). *InlF*, *vip* and *lapB* were absent in few infection and environment-associated STs of lineage II and lineage III (Table S2). Previously described virulence-attenuating point mutations in *plcA* or *plcB* were identified in various STs, independently whether the source of isolation was clinical or environmental^41^ (Table S2). *inlA* mutations leading to InlA truncations, which are commonly found in food isolates^26^,^42^,^43^,^44^,^45^, were not present in any ruminant-associated or environmental isolate. Similarly, mutations of *prfA* that have been associated with hypovirulence were not identified in any of the isolates^46^. Phylogenetic analysis with both the concatenated nucleotide and amino-acid sequences clustered isolates according to their lineage or ST, but not according to their source of isolation (Fig. S2). Diversity varied considerably between virulence-associated genes with the number of alleles ranging between 3 (*hfq*) and 29 (*ctaP*) resulting in 1 (Hfq, SigB) to 25 (InlJ) protein sequences (Table S2). For example, *hfq* had only six synonymous polymorphic sites resulting in three alleles correlating with the three phylogenetic lineages (Fig. S3) but encoding for a single protein sequence conserved across lineages (Fig. 4a). In contrast, *actA* had 26 alleles with 128 non-synonymous polymorphic sites resulting in 21 different protein sequences (Fig. S3, Fig. 4b). Gene sequences of *actA*, *ctaP*, *lapB*, *p60* and *pycA* were ST specific with the exception of STs belonging to CC1, which showed a striking conservation of these genes sharing all the same allele (Table S2, Fig. S3). In some STs more than one allele of these virulence-associated genes was present. In the remaining virulence-associated genes, alleles were shared between some or most STs within the same phylogenetic lineage (Table S2). Importantly, STs involved in rhombencephalitis did not share any common virulence-associated gene allele. Analysis of the 45 virulence genes with ClonalFrame^47^ estimated the relative frequency of recombination occurrence versus mutation (ρ/θ) from 0.01 (*mprF*) to 3.91 (*inlA*), respectively (Table S2). In most virulence genes (n = 37), ρ/θ was below 1 indicating that mutation is more frequent than recombination. Only in four virulence-associated genes (*ctpA*, *gap*, *gtcA*, and *inlA*), the recombination rate was estimated more than 2-fold higher than mutation rate and of those, *gtcA* and *inlA* were subjected to the strongest relative effect of recombination versus point mutation (r/m) (Table S2).

## Discussion

*L. monocytogenes* is organized in four phylogenetic lineages with a clonal population structure^7^,^14^. Recently, hypervirulent and hypovirulent clones, which are differently distributed between human clinical infections and food, were distinguished by combining epidemiological, clinical and experimental approaches^7^. Among the various clinicopathological outcomes associated with listeriosis, neurolisteriosis is associated with a high case fatality rate^9^. Here, we provide evidence for the association of *L. monocytogenes* ST1 (or clone CC1) with neurotropism in ruminants by analysing *L. monocytogenes* isolates from clinicopathologically well-defined ruminant infections, from healthy ruminants and from their environment.

Most ruminant-associated isolates belonged to the two phylogenetic lineages I and II, and in line with former studies, lineage IV and III strains were rare^15^,^25^,^26^. We could show that ruminants are potentially exposed to a large diversity of *L. monocytogenes* strains in their immediate environment, yet not all of these strains are found in clinical infections. We observed that although isolates from ruminant clinical infections belong to both major lineages I and II, lineage I strains are highly prevalent in clinical infections, confirming previous studies in humans and ruminants (Figs 1 and 2)^7^,^24^,^48^. This was particularly true for isolates from neurolisteriosis that strongly clustered in ST1 (CC1) of lineage I. This ST was predominant in and significantly associated with rhombencephalitis suggesting that ST1 has an increased neurotropism in ruminants. Additionally, rhombencephalitis isolates were highly prevalent in ST4 (CC4) and ST412 (CC412), but a significant association was not observed. Bovine macrophage infection assays indicated that STs 1, 4, and 412 are hyperinvasive and hyper-replicative compared to environment-associated STs. Interestingly, ST412 belongs to lineage II indicating that these features are not restricted to strains from lineage I. Results are in line with the hypervirulence of ST1 reported in a previous study^7^ and may suggest that neurotropism in these STs is determined by their hyperinvasiveness and increased intracellular replication, the mechanisms of which have not yet been clarified. Polymorphisms that have been previously associated with virulence-attenuation were rarely detected in ruminant-associated isolates^26^,^41^. Most of them were present in lineage II strains, and they were sporadically detected in ST1 and ST412, which according to their association with clinical infection represent virulent STs. All ruminant associated strains had a full length InlA. Premature stop codons in *inlA*, which are frequently observed in food isolates^40^,^49^,^50^, were absent in our isolates, which may be linked to the fact that CC9 and 121 that are associated with InlA truncations, were not isolated in our study^7^,^26^,^40^. Hence, given that the ruminant amino acid sequence of the InlA host cell receptor E-cadherin determines permissiveness to *L. monocytogenes* infection^51^ and E-cadherin is expressed in ruminant mucosa and nerves^52^, there may be a role of InlA in the pathogenesis of ruminant listeriosis. Phylogenetic analysis of 45 currently known virulence-associated genes showed that most of these genes clustered according to their ST and clone, but neither to their source of isolation nor to the host species (Fig. S2). Additionally, divergence of most virulence-associated genes was driven by mutation rather than by recombination suggesting co-evolution of virulence associated genes and housekeeping genes and vertical transmission of virulence traits. We did not identify any common allele of a virulence-associated gene shared between STs involved in rhombencephalitis, and horizontal gene transfer between rhombencephalitis-associated STs was not obvious. Altogether these data suggest that these known virulence-associated genes are not determining the differences in neurotropism among *L. monocytogenes* strains. The association of lineage I and notably ST1 with neurolisteriosis was particularly strong in cattle (Fig. 1), while in small ruminants neurolisteriosis was caused by a variety of STs from lineages I and II suggesting increased susceptibility of small ruminants to develop neurolisteriosis upon infection with diverse *L. monocytogenes* strains. This interpretation is supported by the high prevalence and mortality and by the frequently fulminant disease and pathology of listeriosis in small ruminants compared to cattle^3^,^6^. Hence, our data may suggest specific virulence mechanisms of ST1 that become relevant in the pathogenesis of cattle rhombencephalitis. ST1 was rarely detected in the ruminant environment possibly suggesting that ST1 is not well adapted to the farm environment. However, as there might be a bias due to small sample size of environmental isolates, this finding needs to be confirmed by analysing larger numbers of isolates.

Hypervirulent CC1 and CC4 belong to the predominant clones in human neurolisteriosis, and CCs identified in ruminant infections partially overlapped with those of human infections in Switzerland and France^7^,^40^ (Fig. 2b) emphasizing similarities of human and animal neurolisteriosis including common mechanisms of host-pathogen interactions in these host species. Nonetheless, the relative prevalences of CCs are distinct between human and ruminant neurolisteriosis. Although prevalences of ruminant-associated CCs might be biased due to the relatively small sample size compared to human studies, these results indicate possible differences in pathogenesis, ecology, niche adaptation and transmission between CCs. Notably the high prevalence of ST1 and the very low prevalence of CC6 in ruminant neurolisteriosis remain striking (Fig. 2)^7^,^40^,^53^. They may be related to phenotypic differences in ruminant and human neurolisteriosis. In ruminants, the cardinal pathology of neurolisteriosis is rhombencephalitis, a brainstem encephalitis that occurs following invasion via cranial nerves^6^,^31^,^54^. In contrast to cattle, human patients are affected by various forms of neurolisteriosis including meningitis, meningoencephalitis, rhombencephalitis and brain abscesses^55^,^56^, of which meningitis and meningoencephalitis clearly prevail^57^,^58^. Hence, future studies should address a potential association of STs/CCs with neurolisteriosis subtypes in humans and whether there are commonalities between the CC1 infection mechanisms of rhombencephalitis in cattle and CNS infection in humans. Host differences such as prevalence of comorbidities, feeding habits and immune response might contribute to the divergence in relative prevalences of clones between humans, small ruminants and cattle^7^,^59^,^60^. Both CC220 and CC412 were abundant in the ruminant-associated isolates from various sources including clinical infections, but have been isolated less frequently from human infections^7^,^40^ (Fig. 2) suggesting an association of these CCs to ruminant species or a specific niche adaptation. STs 37 and 399 were significantly associated with ruminant environment and ruminant faeces. While ST37 has been identified in food processing plants and environment^49^,^50^,^61^ and was responsible for a Belgian listeriosis outbreak linked to pasteurized cheese^62^, ST399 has been only rarely reported until now and at low frequencies (0.3%)^25^. On the other hand, CCs highly prevalent in human infection including CC 2, 3, 5, 6, 7, 29 and 155^7^,^40^ (Fig. 2) were strikingly less prevalent in ruminant-associated isolates. Remarkably, CCs 2 and 6 that are associated with human infection and are hypervirulent in a humanized mouse model^7^, were rarely found in ruminant associated isolates. Similarly, CCs that are highly prevalent in food and food processing plants such as CC9 and CC121 were not represented in our isolates^7^,^50^,^63^,^64^ (Fig. 2) supporting evidence of specific niche adaptation and increased ability to contaminate food, but not farm environments.

To conclude, we provide evidence for ST1-associated hyperinvasiveness, increased intracellular replication and neurotropism of *L. monocytogenes* in ruminants. These findings should be considered when studying molecular mechanisms of host-pathogen interactions and epidemiology in rhombencephalitis. The determination of ST-associated virulence and ecology in *L. monocytogenes* will improve surveillance and control of listeriosis.

## Material and Methods

### Clinical isolates

A total of 187 clinical isolates from ruminant cases were included in the study. Isolates were collected in Switzerland and UK during surveillance and diagnostic activities between 1996 and 2015 (Table S1). Cases of rhombencephalitis (n = 140, cattle = 39, goats = 31, sheep = 70) were defined as such when *L. monocytogenes* was isolated from the brainstem of animals with neurological signs and histopathologically confirmed rhombencephalitis^6^. In abortion cases (n = 33, cattle = 23, goats = 3, sheep = 7), *L. monocytogenes* was isolated from the placenta and/or the foetus. Gastroenteritis cases (sheep = 8) were defined as cases with diarrhoea and pathologically confirmed neutrophilic gastroenteritis^65^, in which *L. monocytogenes* was isolated from the gastrointestinal content. Mastitis cases (n = 6, cattle = 5, goats = 1) were defined by the isolation of *L. monocytogenes* from an udder quarter.

### Environmental isolates

Based on sample size calculations for the detection of differential ST distribution between clinical and environmental isolates (Text. S1) a total of 61 isolates from the direct ruminant environment (n = 56) and faeces of healthy ruminants (n = 5) were included in this study. These isolates originated from cattle (n = 16) and small ruminant (n = 16) farms located in Switzerland (n = 27), Germany (n = 4) and Italy (n = 1), (Table S1).

### Multilocus-sequence-typing (MLST)

MLST is based on the sequence determination of loci in the seven house-keeping genes *abcZ*, *bglA*, *cat*, *dapE*, *dat*, *ldh* and *lhkA*^26^,^27^. Each unique nucleotide sequence is assigned to an arbitrarily defined allele number, and the combination of allele numbers at the seven loci defines the allelic profile or ST. These data can be retrieved at the web-based MLST database from Institut Pasteur (http://bigsdb.web.pasteur.fr/listeria/).

To perform MLST, genomic DNA of all isolates was obtained with the guanidium thiocyanate-phenol-chloroform extraction method^66^. Hundred-twenty-two isolates were subtyped by conventional MLST as previously described^27^. Briefly, the seven housekeeping genes were amplified by PCR and the obtained PCR products were sequenced using universal sequencing primers^26^. Allelic profiles and corresponding STs were determined with the BioNumerics software (Version 7.1, Applied Maths, Austin, TX). With the availability of next generation sequencing technologies we moved on to *in silico* MLST following library preparation and genomic DNA sequencing using either an Illumina HiSeq 2000 or 3000 platform (193 isolates) or an Illumina MiSeq platform (35 isolates) at the Institute of Genetics, Vetsuisse Faculty, University of Bern. The obtained data were assembled using Velvet 1.2.10: algorithms for de novo short read assembly using Bruijn graphs^67^ and housekeeping gene allele numbers and the respective ST were determined with a specifically created workflow in Geneious R7 (http://www.geneious.com).

Finally, all MLST data were entered into the BioNumerics software in order to construct a minimum spanning tree (MST) and to identify CCs and evolutionary lineages. MSTs were produced using default settings. Bar plots with the most important CCs of ruminant isolates and of human clinical cases of Switzerland (n = 93) and France (n = 2304) from previous studies^7^,^40^ were constructed to compare their distribution. We used CCs for these barplots to facilitate comparisons with previous studies. The name of the CC corresponds to the name of the most important ST in this CC.

### Multilocus variable number of tandem repeats analysis (MLVA)

Hundred-eighty-three *L. monocytogenes* isolates from ruminant rhombencephalitis cases (n = 179) and abortion cases (n = 4) had been subtyped by MLVA in a previous study^23^. Additional isolates from abortion cases (n = 20), mastitis cases (n = 5), gastroenteritis cases (n = 8), rhombencephalitis cases (n = 2) and one environmental isolate were submitted to MLVA according to the protocol described previously^23^. Briefly, five bacterial colonies grown on tryptic soy agar were lysed (Qiagen, Mericon DNA Bacteria Plus Kit No. 69534) and used for PCR. For each isolate, eight loci were analysed in four multiplex PCR reactions (Qiagen Multiplex PCR Kit No. 206143) combining two primer loci each. Primers were the same as in ref. 23. The size of the PCR products was determined with the Agilent DNA 1000 Kit in an Agilent 2100 Bioanalyzer (Agilent Technologies, Waldbronn, Germany). The number of repeats in each locus was determined according to Sperry *et al.*^68^. Missing products were registered as “zero alleles” to facilitate MLVA type and cluster analysis. MLVA data were uploaded into the BioNumerics software (Version 7.1, Applied Maths, Austin, TX). A MST was created from a total of 219 *L. monocytogenes* isolates to identify complexes that were defined to contain strains differing in a maximum of two MLVA loci. The MST was produced using a maximum number of N-locus variants (N = 1) with a weight of 10000. MLVA complexes were identified in the MLST-based MST and vice versa (Fig. S1).

### Serotyping

For two isolates the molecular serovar group type was determined using the PCR based protocols as previously described^69^. For 226 isolates with available NGS data, the serovar group type was determined *in silico* using a specifically created workflow in Geneious R7 (http://www.geneious.com) with the primers of ref. 69.

### Gentamicin protection assay

The BoMac cell line was cultured in Dulbecco’s modified eagle medium (DMEM; Life Technologies, Zug, Switzerland) supplemented with 10% fetal calf serum (FCS; Bioswisstec, Schaffhausen, Switzerland) and 100 U/ml penicillin/streptomycin (Life Technologies). Cells were seeded (3 × 10^5^ cells/ml) and grown to confluency in 24-well plates in the above mentioned mediums, without antibiotics. Cells were then starved for 1 h in medium without FCS, followed by the infection with a panel of six representative *L. monocytogenes* cattle isolates at a MOI of 10: ST1, ST4 and ST412 isolated from rhombencephalitis; ST1, ST18 and ST37 isolated from environment. One hour post infection, the inoculum was removed, cells were washed with phosphate buffered saline (PBS) and covered with medium containing 10% FCS and 50 μg/ml gentamicin (Sigma-Aldrich) to inhibit extracellular growth of *L. monocytogenes*. At indicated time points post infection, cells were washed with PBS and lysed with 0.5% Triton-X100 (Sigma-Aldrich) to harvest CFUs. Serial dilutions were plated on brain heart infusion (BHI) agar and CFUs were quantified. For testing of invasiveness, CFUs were harvested at 2 h and for testing of intracellular replication, CFUs were harvested at 4 h, 8 h and 24 h post infection^46^. Three independent experiments were performed in triplicates, and results were normalized to the inoculum.

### Virulence-associated gene analysis

The nucleotide and amino acid sequences of 45 previously described virulence factors known to be involved in the virulence of *L. monocytogenes*^39^ were compared between 94 isolates with NGS data of sufficient quality (Table S1). The 94 isolates belonged to 25 STs and included 35 rhombencephalitis isolates (cattle = 15; small ruminants = 20), seven clinical non-encephalitic isolates (cattle = 5; small ruminants = 2) and 52 environmental isolates (cattle = 33; small ruminants = 19). The data was analyzed for mutations associated with attenuated virulence described in previous studies^23^,^26^,^41^. Using the sequences of the 45 virulence genes from EGD-e (GenBank accession NC_003210), the corresponding genes were extracted from our 94 genome sequences after a MAFFT v7. 222 alignment^70^. This was then followed by the construction of neighbour-joining trees based on the nucleotide level with synonymous and non-synonymous mutations and on the amino acid level with Geneious R7 (http://www.geneious.com). Additionally, neighbour-joining trees were built based on the concatenated nucleotide and amino acid sequences of all virulence factors following a MAFFT v7. 222 alignment. Recombination events in the 45 virulence genes were computed using the ClonalFrame v1.1^47^ and ML^71^ as described in ref. 72 in five individual runs. The mean of the five runs was calculated for each virulence gene, and the 95% CI of these means was determined.

### Statistical analysis

The R software (R Core Team (2014). R: A language and environment for statistical computing. R Foundation for Statistical Computing, Vienna, Austria. ISBN 3-900051-07-0, URL http://www.R-project.org/) was used to compare the frequency of different STs in different groups of isolates with the Fisher’s exact test. As not all variables were normally distributed using the Shapiro-Wilk test, the non-parametric Kruskal-Wallis test and the Dunn’s multiple comparison post-hoc tests were used to assess significant differences in CFU counts. Differences between groups with a *p-*value < 0.05 were considered to be statistically significant. Genetic diversity of *L. monocytogenes* was estimated by the Simpson index of diversity^73^, which calculates the probability that two isolates belong to different STs and the 95% CI of the analyzed groups was determined^74^.

### Accession numbers

The sequencing data of the 94 isolates used in the virulence associated gene analysis were submitted to the ENA (European Nucleotide Archive) database (Data Set. S1).

## Additional Information

**How to cite this article**: Dreyer, M. *et al.* *Listeria monocytogenes* sequence type 1 is predominant in ruminant rhombencephalitis. *Sci. Rep.* **6**, 36419; doi: 10.1038/srep36419 (2016).

**Publisher’s note:** Springer Nature remains neutral with regard to jurisdictional claims in published maps and institutional affiliations.

## Supplementary Material

Supplementary Information

Supplementary Dataset 1

## Figures and Tables

**Table 1:** Prevalence of phylogenetic lineage I and II in rhombencephalitic, non-encephalitic and environmental isolates.

Source	No. (%) of isolates								
	Rhombencephalitis			Non-encephalitic infections			Environment/faeces		
	Total	Cattle	Small ruminants	Total	Cattle	Small ruminants	Total	Cattle	Small ruminants
Total	140	39	101	47	28	19	61	36	25
Lineage I	101 (72)***	33 (85)***	68 (67)*	18 (38)	7 (25)	11 (58)	22 (36)	13 (36)	9 (36)
Lineage II	39 (28)	6 (15)	33 (33)	29 (62)**	21 (75)	8 (42)	37 (61)***	23 (64)*	14 (56)*

Fisher’s test: *p-value < 0.05; **p-value < 0.01; ***p-value < 0.001.
