# Supplementary material for: Listeria monocytogenes sequence type 1 is predominant in ruminant rhombencephalitis
Source: Sci Rep. 2016 Nov 16;6:36419. doi: 10.1038/srep36419 (PMC5111077; doi:10.1038/srep36419)
Supplement: Supplementary Information [file srep36419-s1.pdf]

Listeria monocytogenes sequence type 1 is predominant in ruminant rhombencephalitis

Margaux Dreyer, [a, b] Lisandra Aguilar-Bultet,[b, c] Sebastian Rupp,[a, b] Claudia Guldimann, [a, b#] Roger Stephan, [d] Alexandra Schock, [e] Arthur Otter, [f] Gertraud Schüpbach, [g] Sylvain Brisse, [h] Marc Lecuit, [i, j, k, l] Joachim Frey, [c] Anna Oevermann [a]*

Division of Neurological Sciences, Department of Clinical Research and Veterinary Public Health, Vetsuisse Faculty, University of Bern, Switzerland [a]; Graduate School for Cellular and Biomedical Sciences, Theodor Kocher Institute, University of Bern, Switzerland [b]; Institute of Veterinary Bacteriology, Department of Infectious Diseases and Pathobiology, University of Bern, Switzerland [c]; Institute for Food Safety and Hygiene, University of Zurich, Switzerland [d]; Department of Pathology, AHVLA Lasswade, Pentlands Science Park, Bush Loan, Penicuik Midlothian, United Kingdom [e]; AHVLA Shrewsbury Kendal Road, Harlscott, Shrewsbury, Shropshire, United Kingdom [f]; Veterinary Public Health Institute, University of Bern, Switzerland [g]; Institut Pasteur, Microbial Evolutionary Genomics, Paris, France [h]; Institut Pasteur, Biology of Infection Unit, Paris, France [i]; Inserm U1117, Paris, France [j]; French National Reference Center and WHO Collaborating Center *Listeria*, Institut Pasteur, Paris, France [k]; Paris Descartes University, Sorbonne Paris Cité, Division of Infectious Diseases and Tropical Medicine, Necker-Enfants Malades University Hospital, Institut Imagine, Paris, France [l]

[#] Present address: Claudia Guldimann, Institute for Food Safety and Hygiene, University of Zurich, Switzerland

* Address correspondence to Anna Oevermann, anna.oevermann@vetsuisse.unibe.ch

Text. S1:

On-farm distribution of *Listeria monocytogenes* and risk factor analysis

Listeria monocytogenes is frequently present in ruminant farms

We estimated a sample size of 60 environmental isolates, to detect an Odds Ratio of 4 in the differential distribution of sequence types (STs) between ruminant clinical and non-clinical isolates with a power of 80 % and a confidence of 95 %. For this analysis the Win Episcope 2.0 software [1] was used. Thus, a total of 32 ruminant farms were sampled in a cross sectional study (Table. S3) from January 2014 to May 2014 and from October 2014 to May 2015. Case farms were defined based on the occurrence of confirmed ruminant listeriosis cases during the last five years prior to sampling. Control farms were defined based on the absence of clinical signs suggestive of listeriosis in the animal population over the last five years. Case farms were selected randomly among the available farms in our database and control farms of similar herd size were selected from the same database. Sixteen farms each primarily housed cattle (case farms n=6, control farms n=10) or small ruminants (sheep n =10, goats n =6; case farms n=12, control farms n=4). One farm was sampled during an ongoing listeriosis outbreak and three sampled farms endured outbreaks up to three months prior to sampling. Of three sampled farms we obtained brain isolates of animals, which died due to listeriosis on these farms. Twenty-seven farms were located in Switzerland, four in Germany and one in Italy.

During each farm visit six environmental samples and one faecal sample pool were collected as described in a previous study [2]. Briefly, 25 g of soil, silage and commodity feeds of 10 randomly chosen areas were collected, as well as faeces of 10 to 30 randomly selected animals during defecation. Additionally, swabs using sterile gauze pads moistened with 0.9 % NaCl were taken

from feed bunks, water tanks and floors of 10 randomly chosen areas. All samples were immediately transported to the lab and processed within the next 24 h.

As previously described [2], *Listeria* (*L.) monocytogenes* was isolated by a primary enrichment of pooled samples (25 g or 10 gauze pads) in 225 ml of the Oxoid Novel Enrichment Broth (Oxoid, Ltd., Basingstoke, United Kingdom), followed by a secondary enrichment with a 1:10 dilution of the primary enrichment. The sensitivity of the two-step enrichment procedure to isolate *L. monocytogenes* from pooled environmental samples was tested previously and shown to be 1 cfu/ml of broth [2]. Broth of both the primary and secondary enrichment was streaked on Brilliance Listeria and Palcam agar plates (Oxoid, Ltd, Basingstoke, United Kingdom). Per plate, up to four single colonies with typical *L. monocytogenes* morphology were transferred to Tryptic Soy Agar (TSA) plates containing 5% sheep blood (BD, Becton Dickinson and Company, Sparks, U.S.A) for species confirmation with the Matrix-Assisted Laser Desorption Ionization-Time Of Flight Mass Spectrometry (MALDI-TOF-MS, Bruker MALDI-TOF BiotyperTM110) analytical tool according to Barbuddhe et al. [3]. The presence of a peak at either 5599 Da or 5593 Da enabled the prediction of lineage I and lineage II or III, respectively. Single colonies of *L. monocytogenes* were transferred into glycerol and stored at – 80 °C until further use. To avoid redundancy, only one colony per enrichment and selective medium was further analyzed by MLST, if all colonies from the same date of sampling, farm and environmental source were of the same lineage. If MALDI-TOF-MS analysis indicated the presence of colonies from different phylogenetic lineages, colonies of both lineages were analyzed. In total, twenty-two lineage I isolates and 39 isolates from lineage II or III, as identified by MALDI-TOF-MS, were further subtyped by MLST.

L. monocytogenes was detected in the majority of farms (66 %, n =21), but only in 21 % (n =47) of the environmental and faecal samples, with zero to four positive samples per farm (Table. S3).

Sixty-one *L. monocytogenes* isolates were obtained from the 47 positive samples. Most frequently, *L. monocytogenes* was isolated from soil (37%, n =12) and floor (34 %, n =11). Additionally, *L. monocytogenes* was present in water tanks (22 %, n =7), feed bunk swabs (22 %, n =7), faeces (16 %, n =5) and commodity feed (12 %, n =4). In contrast, *L. monocytogenes* was detected in silage only on a single farm. The frequency of *L. monocytogenes* detection in farm samples was significantly higher in cattle farms (n =30) than in small ruminant farms (n =17; Fisher's test, p-value < 0.05). However, this effect was not observed when the frequency in a single environmental source was compared between cattle and small ruminant farms, possibly due to the small sample size per source. Finally, the frequency of *L. monocytogenes* detection was similar between control and case farms at the farm and sample level (Table. S4). In two of three farms, in which a clinical isolate was available, the ST of the clinical isolate matched the ST of the environmental and faecal isolate (ST1 and ST4). In four out of five farms where *L. monocytogenes* was isolated from faeces, the faecal isolate was of the same ST as the environmental isolate (ST4, ST70, ST399 and ST451).

The prevalence of *L. monocytogenes* in the farm environment has only been addressed by few studies [4-6]. We detected *L. monocytogenes* in the majority of ruminant farms, but in the minority of samples. The prevalence is consistent with previous studies [7,8] challenging the view that *L. monocytogenes* is ubiquitous [9]. The prevalence was significantly higher in cattle farms than in small ruminant farms confirming the view that ecology of *L. monocytogenes* differs between cattle and small ruminant farms [8]. However, no difference in prevalence was observed between case and control farms. *L. monocytogenes* was most frequently detected in soil and matching STs were identified in soil and other materials suggesting that farm soil could represent a contamination source for other materials (Table. S4). In contrast, and in accordance with a previous study [10] *L. monocytogenes* was rarely detected in silage, which has been inculpated to be

the main source of infection in farm ruminants [8,11-13]. This may be related to the good quality of most of the analyzed silage in this study (data not shown). The prevalence in faecal samples was low (16% overall) but in the range of previously reported data [14-16], challenging the view that ruminants act as amplification hosts and that faecal shedding is linked to silage feeding as silage was fed on most farms [17]. However, in all five farms where faecal samples were positive either feed bunk swabs, hay or water samples were also positive with the same ST, suggesting that faecal shedding is linked to contamination of feed and water.

The presence of *L. monocytogenes* is likely linked to farm management practices

Feeding and farm management practices were recorded in an observational cross sectional study based on a standardized questionnaire. These multiple choice questionnaires were handed out in each of the 32 visited ruminant farms and comprised a total of 100 different potential risk factors.

For identification of risk and preventive factors univariable logistic regression analysis was performed. The outcome was defined as the presence or absence of *L. monocytogenes* at the farm level (bivariable yes/no) and exposure was defined as the presence or absence of a given risk factor (bivariable yes/no). All risk factors presenting a *p*-value up to 0.20 were further analyzed in the multivariable logistic regression analysis. This was performed for each set of factors with a biological relevance, using a backward elimination procedure (removing the less significant factor) until all variables in the model were significant (p-value <0.05). Odds ratios, confidence intervals and the goodness of fit (likelihood ratio test) of each model were assessed, as well as searching for interaction terms, correlations and confounders. All statistics were produced with the R software (*R Core Team (2014). R: A language and environment for statistical computing. R Foundation for Statistical Computing, Vienna, Austria. ISBN 3-900051-07-0, URL* http://www.R-project.org/).

With the univariable logistic regression, 24 factors had a *p*-value < 0.20 and five of those were significant (deep straw housing: *p*-value < 0.05, OR = 0.13; frequency of straw removal lower than once per week: *p*-value < 0.05, OR = 0.16; bale silage: *p*-value < 0.05, OR = 11.42; maize as main silage component: *p*-value < 0.01, OR = 11.25; grass/maize mix as main silage component: *p*-value < 0.01, OR = 20.0). In the multivariable logistic regression analysis of feed related factors (n = 13), no factor remained significant after all eliminations. In the second multivariable logistic regression analysis of 16 barn-related factors, two factors emerged as significant preventive factors without interaction or correlations (0.25; goodness of fit: 0.004): deep straw housing (coefficient = -2.2, SE = 0.99, 95% CI = -4.46 to 0.45, *p*-value < 0.05, OR = 0.10, 95% OR CI = 0.01 to 0.63) and frequency of straw removal lower than once per week: coefficient = -2.0, SE = 1.0, 95% CI = -4.46 to 0.45, *p*-value < 0.05, OR = 0.12, 95% OR CI = 0.01to 0.63).

This analysis indicates that the presence of *L. monocytogenes* is likely linked to farm management practices. Deep straw housing significantly reduced the isolation of *L. monocytogenes* compared to straw, sand and sawdust. Similarly, a frequency of straw removal lower than once per week reduced the isolation of *L. monocytogenes* when compared to daily and weekly cleaning. In deep straw bedding heat is produced due to composting of the deeper layers, and the microbiome of straw-excrement mixtures is similar to the microbiome of maturing compost, wherefore growth conditions might be suboptimal for *L. monocytogenes* with the presence of competing microbiota [18]. Indeed, *Firmicutes* are much less abundant than other bacteria in deep straw litter compared to other housing systems [19]. The low frequency of cleaning may be an additional preventive factor as the composting effect in deep litter may require some time to occur. Furthermore, in these settings cleaning procedures might be more thoroughly performed.

Figure. S1:

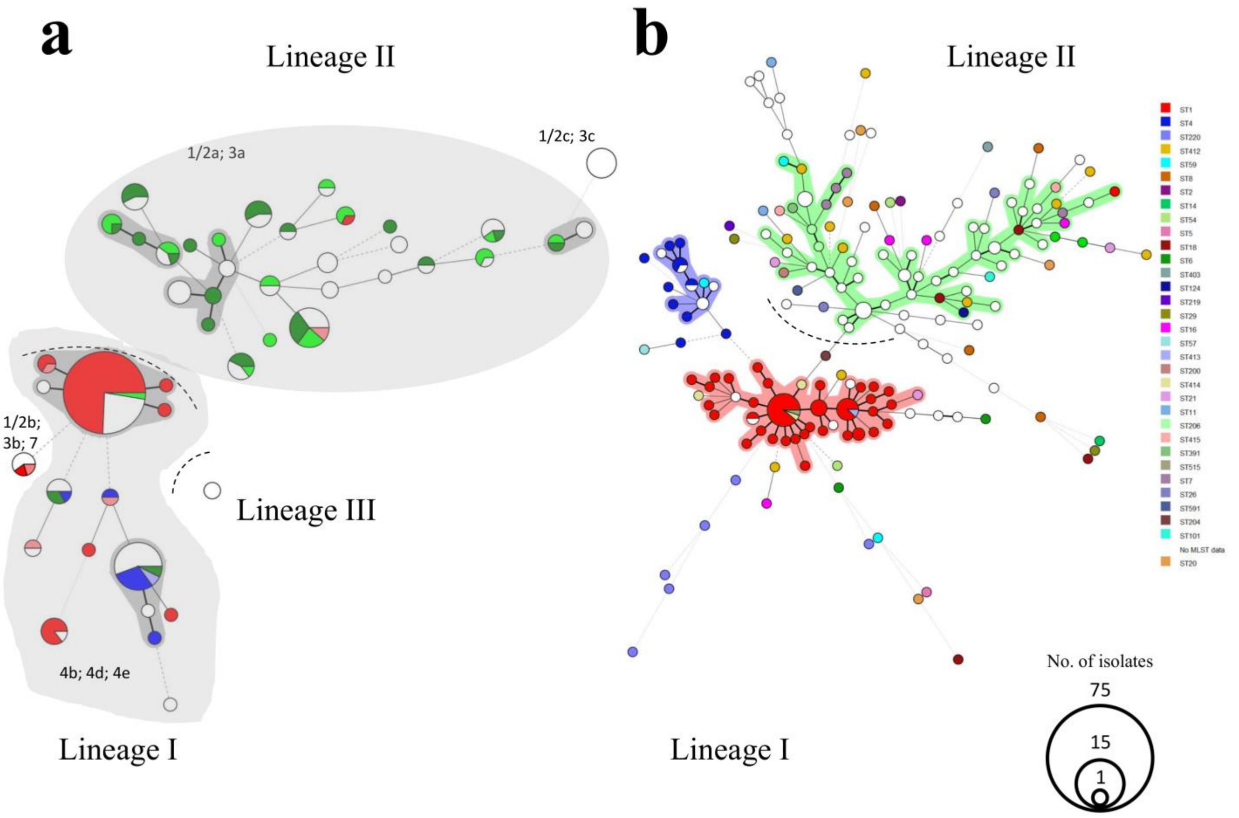

Multilocus sequence typing (MLST) and multilocus variable number of tandem repeat analysis (MLVA) comparisons. a: Minimum spanning tree (MST) of 248 *L. monocytogenes* isolates comparing MLST analysis with MLVA data. Circles represent sequence types (STs) as in Fig.1, whose size corresponds to the number of isolates present in each ST. The lines between different STs represent phylogenetic relationships, as in Fig. 1. Grey zones surrounding multiple STs, represent clonal complexes (CCs) containing STs with a single mismatch in the seven loci. The three phylogenetic lineages are indicated on the tree. Serotypes are indicated next to the corresponding circles. Isolates of MLVA complex A are represented in red (n =66) and correspond largely to CC1, isolates linked to complex A are represented in pink (n =12).

Complex B isolates are indicated in blue (n =11), isolates linked to complex B in purple (n =2). This complex corresponds largely to CC4 in the MLST. Complex C isolates are indicated in dark green (n =32) and isolates linked to complex C in light green (n =23). These isolates correspond to lineage II in MLST. Isolates without MLVA data are coloured white (n =77) [20]. b: MST based on MLVA analysis of 221 clinical *L. monocytogenes* isolates of ruminants and humans and food isolates from a previous study [20] and of 38 isolates from this study. The size of the circle is proportional to the number of isolates belonging to the MLVA type. MLST data available from 183 isolates are integrated for comparison. The colours of each circle correspond to one of the 33 STs identified by MLST. The length of the branches represents genetic distances between two neighbouring types. Heavy black lines joining the circles indicate single locus differences between MLVA types, light black lines indicate differences in two or three loci and light dotted lines differences in 4 or more loci. Coloured zones surrounding the circles delineate the clonal complexes, which were created based on the maximum neighbour distance of changes at two loci and the minimum size of five types. Red: MLVA complex A; violet: MLVA complex B; green: MLVA complex C.

Figure. S2:

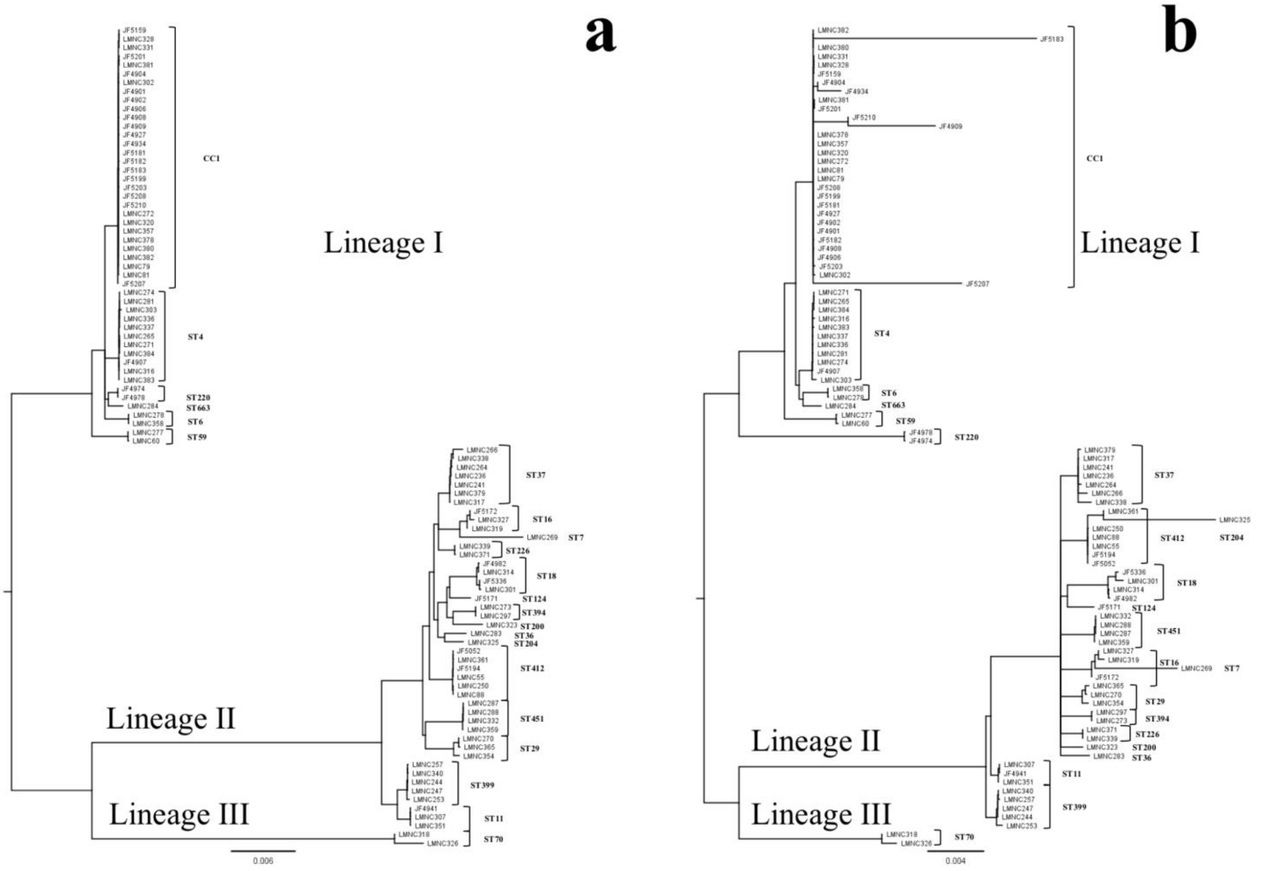

Dendograms showing the phylogenetic divergence of 94 *L. monocytogenes* isolates from different ruminant sources based on concatenated sequences of 45 virulence-associated genes. a: alignment based on nucleotides. b: alignment based on amino acids. Corresponding sequence types (STs) or clonal complexes (CCs), as well as the three evolutionary lineages are given next to the isolates. The linkage distance bar is inserted beneath the tree.

Figure. S3:

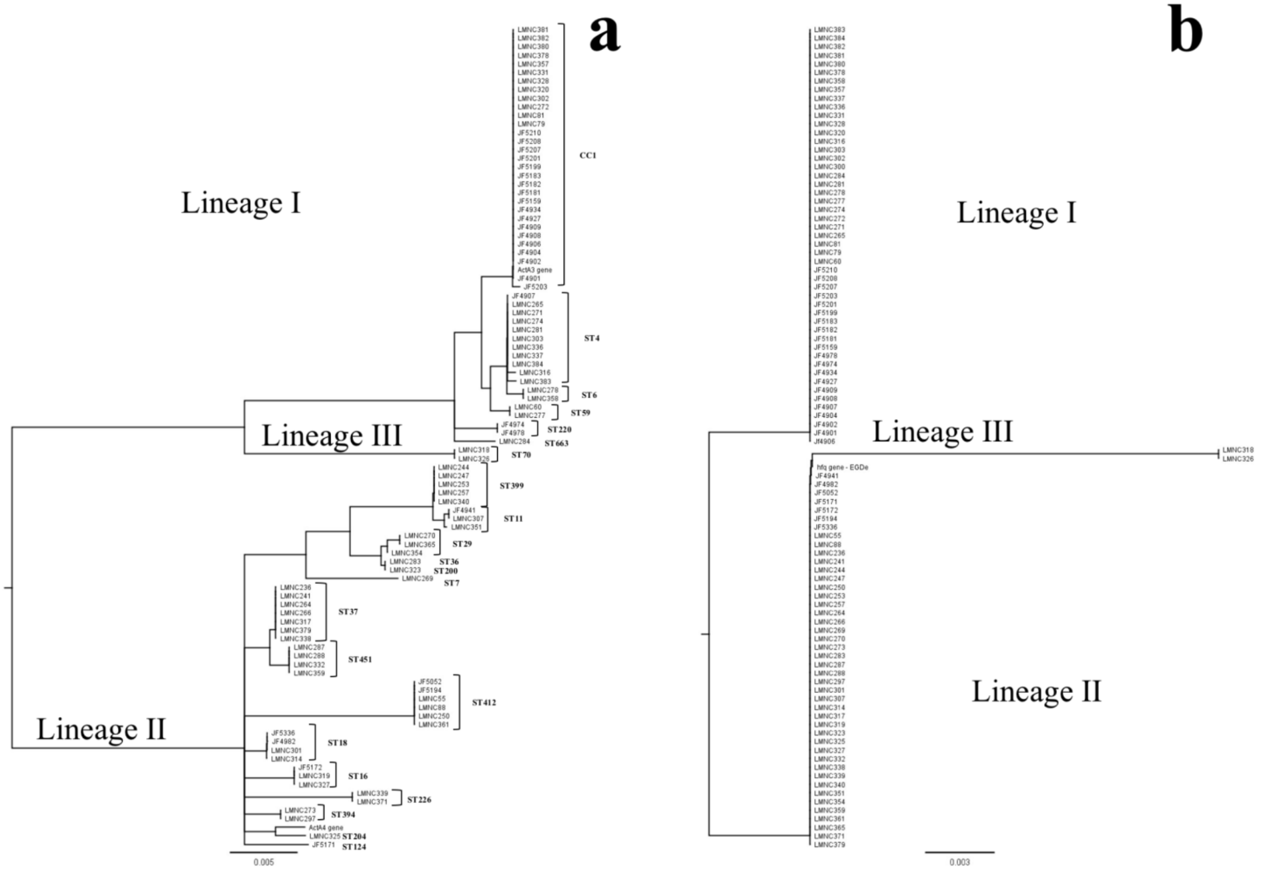

Dendograms showing genetic relationships of 94 *L. monocytogenes* isolates from different ruminant sources. a: alignment based on nucleotides of *actA*. b: alignment based on nucleotides of *hfq*. Corresponding sequence types (STs) or clonal complexes (CCs) obtained with MLST analyses are given next to the isolates, as well as the three evolutionary lineages. The linkage distance bar is inserted beneath the tree

References

1 Thrusfield, M., Ortega, C., de Blas, I., Noordhuizen, J. P. & Frankena, K. WIN EPISCOPE 2.0: improved epidemiological software for veterinary medicine. *The Veterinary record* **148**, 567-572 (2001).

2 Dreyer, M., Thomann, A., Bottcher, S., Frey, J. & Oevermann, A. Outbreak investigation identifies a single *Listeria monocytogenes* strain in sheep with different clinical manifestations, soil and water. *Veterinary microbiology* **179**, 69-75, doi:10.1016/j.vetmic.2015.01.025 (2015).

3 Barbuddhe, S. B. *et al.* Rapid identification and typing of listeria species by matrix-assisted laser desorption ionization-time of flight mass spectrometry. *Applied and environmental microbiology* **74**, 5402-5407, doi:10.1128/AEM.02689-07 (2008).

4 Linke, K. *et al.* Reservoirs of listeria species in three environmental ecosystems. *Applied and environmental microbiology* **80**, 5583-5592, doi:10.1128/AEM.01018-14 (2014).

5 Parisi, A. *et al.* Amplified Fragment Length Polymorphism and Multi-Locus Sequence Typing for high-resolution genotyping of *Listeria monocytogenes* from foods and the environment. *Food microbiology* **27**, 101-108, doi:10.1016/j.fm.2009.09.001 (2010).

6 Ruckerl, I. *et al. L. monocytogenes* in a cheese processing facility: Learning from contamination scenarios over three years of sampling. *International journal of food microbiology* **189**, 98-105, doi:10.1016/j.ijfoodmicro.2014.08.001 (2014).

7 Fox, E., Hunt, K., O'Brien, M. & Jordan, K. *Listeria monocytogenes* in Irish Farmhouse cheese processing environments. *International journal of food microbiology* **145 Suppl 1**, S39-45, doi:10.1016/j.ijfoodmicro.2010.10.012 (2011).

8 Nightingale, K. K. *et al.* Ecology and transmission of *Listeria monocytogenes* infecting ruminants and in the farm environment. *Appl.Environ.Microbiol.* **70**, 4458-4467 (2004).

9 Haase, J. K. *et al.* The ubiquitous nature of *Listeria monocytogenes* clones: a large-scale Multilocus Sequence Typing study. *Environmental microbiology* **16**, 405-416, doi:10.1111/1462-2920.12342 (2014).

10 Mohammed, H. O. *et al.* Identification of potential on-farm sources of *Listeria monocytogenes* in herds of dairy cattle. *Am.J.Vet.Res.* **70**, 383-388 (2009).

11 Ho, A. J., Ivanek, R., Grohn, Y. T., Nightingale, K. K. & Wiedmann, M. *Listeria monocytogenes* fecal shedding in dairy cattle shows high levels of day-to-day variation and includes outbreaks and sporadic cases of shedding of specific L. monocytogenes subtypes. *Prev.Vet.Med.* **80**, 287-305 (2007).

12 Schoder, D. *et al.* Important vectors for *Listeria monocytogenes* transmission at farm dairies manufacturing fresh sheep and goat cheese from raw milk. *Journal of food protection* **74**, 919-924, doi:10.4315/0362-028X.JFP-10-534 (2011).

13 Wiedmann, M. *et al.* Ribotype diversity of *Listeria monocytogenes* strains associated with outbreaks of listeriosis in ruminants. *J.Clin.Microbiol.* **34**, 1086-1090 (1996).

14 Esteban, J. I., Oporto, B., Aduriz, G., Juste, R. A. & Hurtado, A. Faecal shedding and strain diversity of *Listeria monocytogenes* in healthy ruminants and swine in Northern Spain. *BMC.Vet.Res.* **5:2.**, 2 (2009).

15 Ho, A. J., Lappi, V. R. & Wiedmann, M. Longitudinal monitoring of *Listeria monocytogenes* contamination patterns in a farmstead dairy processing facility. *J.Dairy Sci.* **90**, 2517-2524 (2007).

16 Hasegawa, M. *et al.* Prevalence and characteristics of *Listeria monocytogenes* in feces of black beef cattle reared in three geographically distant areas in Japan. *Foodborne pathogens and disease* **11**, 96-103, doi:10.1089/fpd.2013.1616 (2014).

17 Fenlon, D. R., Wilson, J. & Donachie, W. The incidence and level of *Listeria monocytogenes* contamination of food sources at primary production and initial processing. *J.Appl.Bacteriol.* **81**, 641-650 (1996).

18 Erickson, M. C., Liao, J., Ma, L., Jiang, X. & Doyle, M. P. Thermal and nonthermal factors affecting survival of Salmonella and *Listeria monocytogenes* in animal manure-based compost mixtures. *Journal of food protection* **77**, 1512-1518, doi:10.4315/0362-028X.JFP-14-111 (2014).

19 Kumari, P. & Choi, H. L. Manure removal system influences the abundance and composition of airborne biotic contaminants in swine confinement buildings. *Environmental monitoring and assessment* **187**, 537, doi:10.1007/s10661-015-4759-0 (2015).

20 Balandyte, L., Brodard, I., Frey, J., Oevermann, A. & Abril, C. Ruminant rhombencephalitis-associated *Listeria monocytogenes* alleles linked to a multilocus variable-number tandem-repeat analysis complex. *Appl.Environ.Microbiol.* **77**, 8325-8335 (2011).

Table. S1

Summary of all isolates

Isolates			Species	Type	Country	Serotype	Lineage	Sequence Type (ST)	Method (PCR/MiSeq/HiSeq)	MLVA Complex	Sequence analysis of virulence associated genes
**LMNC ID**	**JF ID**	**Balandyte et al., 2011 ID**									
**LMNC01**	**JF5203***	**L146/2007**	Cattle	Rhombencephalitis	Switzerland	4b; 4d; 4e	1	1	HiSeq	A	Yes
**LMNC022**	**JF5194***	**L127/2007**	Sheep	Rhombencephalitis	Switzerland	1/2a; 3a	2	412	HiSeq	C	Yes
**LMNC023**	**JF5201***	**L142/2007**	Cattle	Rhombencephalitis	Switzerland	4b; 4d; 4e	1	1	HiSeq	A	Yes
**LMNC025**	**JF5208***	**L120/2007**	Cattle	Rhombencephalitis	Switzerland	4b; 4d; 4e	1	1	HiSeq	A	Yes
**LMNC031**	**JF5588**		Sheep	Soil	Switzerland	4b; 4d; 4e	1	4	HiSeq	nd	No
**LMNC039**	**JF5589**		Sheep	Water	Switzerland	4b; 4d; 4e	1	4	HiSeq	B	No
**LMNC047**	**JF4900**	**L102/2007**	Cattle	Rhombencephalitis	Switzerland	4b; 4d; 4e	1	1	HiSeq	A	No
**LMNC048**	**JF4904***	**L108/2007**	Cattle	Rhombencephalitis	Switzerland	4b; 4d; 4e	1	1	HiSeq	A	Yes
**LMNC050**	**JF4908***	**L115/2007**	Cattle	Rhombencephalitis	Switzerland	4b; 4d; 4e	1	1	HiSeq	A	Yes
**LMNC051**	**JF4912**	**L138/2007**	Cattle	Rhombencephalitis	Switzerland	1/2a; 3a	2	412	HiSeq	C	No
**LMNC052**	**JF4914**	**L132/2007**	Goat	Rhombencephalitis	Switzerland	1/2a; 3a	2	20	HiSeq	Linked to C	No
**LMNC053**	**JF4915**	**L148/2008**	Goat	Rhombencephalitis	Switzerland	4b; 4d; 4e	1	1	HiSeq	A	No
**LMNC054**	**JF4918**	**L21/2007**	Goat	Rhombencephalitis	Switzerland	4b; 4d; 4e	1	1	HiSeq	A	No
**LMNC055***	**JF4926**	**L126/2007**	Goat	Rhombencephalitis	Switzerland	1/2a; 3a	2	412	HiSeq	Linked to A	Yes
**LMNC056**	**JF4929**	**L41/2007**	Sheep	Rhombencephalitis	Switzerland	4b; 4d; 4e	1	4	HiSeq	Linked to B	No
**LMNC057**	**JF4930**	**L43/2007**	Sheep	Rhombencephalitis	Switzerland	1/2a; 3a	2	206	HiSeq	C	No
**LMNC058**	**JF4934***	**L2/2007**	Sheep	Rhombencephalitis	Switzerland	4b; 4d; 4e	1	414	HiSeq	A	Yes
**LMNC059**	**JF4939**	**L26/2007**	Sheep	Rhombencephalitis	Switzerland	4b; 4d; 4e	1	1	HiSeq	A	No
**LMNC060***	**JF4942**	**L38/2007**	Sheep	Rhombencephalitis	Switzerland	1/2b; 3b; 7	1	59	HiSeq	C	Yes
**LMNC061**	**JF5150**	**L57/2007**	Sheep	Rhombencephalitis	Switzerland	4b; 4d; 4e	1	1	HiSeq	A	No
**LMNC062**	**JF5157**	**L81/2007**	Cattle	Rhombencephalitis	Switzerland	4b; 4d; 4e	1	1	HiSeq	A	No
**LMNC063**	**JF5164**	**L47/2007**	Goat	Rhombencephalitis	Switzerland	1/2a; 3a	2	11	HiSeq	Linked to C	No
**LMNC064**	**JF5166**	**L42/2007**	Sheep	Rhombencephalitis	Switzerland	4b; 4d; 4e	1	4	HiSeq	B	No
**LMNC065**	**JF5167**	**L44/2007**	Cattle	Rhombencephalitis	Switzerland	1/2a; 3a	2	206	HiSeq	C	No
**LMNC066**	**JF5178**	**O/D1171/06**	Cattle	Abortion	Switzerland	1/2a; 3a	2	101	HiSeq	Linked to C	No
**LMNC067**	**JF5180**	**L77/2007**	Sheep	Rhombencephalitis	Switzerland	4b; 4d; 4e	1	4	HiSeq	B	No
**LMNC069**	**JF5196**	**L122/2007**	Cattle	Rhombencephalitis	Switzerland	4b; 4d; 4e	1	4	HiSeq	Linked to B	No
**LMNC071**	**JF5213**	**L131/2007**	Goat	Rhombencephalitis	Switzerland	1/2a; 3a	2	26	HiSeq	Linked to C	No
**LMNC074**	**JF5191**	**L135/2007**	Cattle	Rhombencephalitis	Switzerland	4b; 4d; 4e	1	1	HiSeq	A	No
**LMNC075**	**JF5186**	**L78/2007**	Sheep	Rhombencephalitis	Switzerland	4b; 4d; 4e	1	54	HiSeq	Linked to A	No
**LMNC076**	**JF4936**	**L13/2007**	Sheep	Rhombencephalitis	Switzerland	4b; 4d; 4e	1	4	HiSeq	B	No
**LMNC077**	**JF5173**	**L157/2008**	Sheep	Rhombencephalitis	Switzerland	4b; 4d; 4e	1	4	HiSeq	B	No
**LMNC078**	**JF5212**	**L97/2007**	Sheep	Rhombencephalitis	Switzerland	1/2a; 3a	2	18	HiSeq	C	No
**LMNC079***	**JF4923**	**L84/2007**	Goat	Rhombencephalitis	Switzerland	4b; 4d; 4e	1	1	HiSeq	Linked to C	Yes
**LMNC081***	**JF4924**	**L87/2007**	Goat	Rhombencephalitis	Switzerland	4b; 4d; 4e	1	1	HiSeq	Linked to C	Yes
**LMNC082**	**JF4913**	**L143/2007**	Cattle	Rhombencephalitis	Switzerland	1/2a; 3a	2	412	HiSeq	Linked to C	No
**LMNC083**	**JF4937**	**L22/2007**	Sheep	Rhombencephalitis	Switzerland	1/2a; 3a	2	16	HiSeq	Linked to C	No
**LMNC084**	**JF4941***	**L36/2007**	Sheep	Rhombencephalitis	Switzerland	1/2a; 3a	2	11	HiSeq	Linked to C	Yes
**LMNC085**	**JF4921**	**L45/2007**	Goat	Rhombencephalitis	Switzerland	1/2a; 3a	2	412	HiSeq	Linked to C	No
**LMNC086**	**JF4940**	**L35/2007**	Sheep	Rhombencephalitis	Switzerland	1/2a; 3a	2	21	HiSeq	Linked to C	No
**LMNC086**	**JF5174**	**L54/2007**	Sheep	Rhombencephalitis	Switzerland	1/2a; 3a	2	8	HiSeq	Linked to C	No
**LMNC087**	**JF5214**	**L86/2007**	Sheep	Rhombencephalitis	Switzerland	1/2a; 3a	2	204	HiSeq	Linked to C	No
**LMNC088***	**JF5590**		Cattle	Silage	Switzerland	1/2a; 3a	2	412	HiSeq	nd	Yes
**LMNC096**		**L18/2007**	Sheep	Rhombencephalitis	Switzerland	4b; 4d; 4e	1	6	HiSeq	Linked to A	No
**LMNC097**		**L162/2008**	Sheep	Rhombencephalitis	Switzerland	4b; 4d; 4e	1	4	HiSeq	B	No
**LMNC121**	**JF5197**	**L96/2007**	Goat	Rhombencephalitis	Switzerland	1/2a; 3a	2	21	HiSeq	A	No
**LMNC129**	**JF5591**		Cattle	Water	Switzerland	4b; 4d; 4e	1	1	HiSeq	nd	No
**LMNC134**	**JF5592**		Cattle	Floor	Switzerland	4b; 4d; 4e	1	5	HiSeq	nd	No
**LMNC137**	**JF5593**		Cattle	Feed bunk	Switzerland	1/2a; 3a	2	412	HiSeq	nd	No
**LMNC139**	**JF4990**		Sheep	Abortion	Great Britain	4b; 4d; 4e	1	220	HiSeq	A	No
**LMNC140**	**JF4978***		Sheep	Enteritis	Great Britain	4b; 4d; 4e	1	220	HiSeq	A	Yes
**LMNC141**	**JF4971**		Cattle	Abortion	Great Britain	nd	1	59	PCR	C	No
**LMNC142**	**JF4977**		Sheep	Enteritis	Great Britain	nd	1	54	PCR	B	No
**LMNC143**	**JF4976**		Cattle	Abortion	Great Britain	1/2a; 3a	2	14	HiSeq	C	No
**LMNC144**	**JF4975**		Cattle	Abortion	Great Britain	nd	1	2	PCR	A	No
**LMNC145**	**JF4974***		Cattle	Abortion	Great Britain	4b; 4d; 4e	1	220	HiSeq	A	Yes
**LMNC146**	**JF4994**		Sheep	Abortion	Great Britain	4b; 4d; 4e	1	220	HiSeq	A	No
**LMNC147**	**JF4991**		Sheep	Abortion	Great Britain	1/2a; 3a	2	403	HiSeq	C	No
**LMNC148**	**JF4973**		Cattle	Abortion	Great Britain	nd	2	8	PCR	C	No
**LMNC149**	**JF4982***		Sheep	Enteritis	Great Britain	1/2a; 3a	2	18	HiSeq	C	Yes
**LMNC150**	**JF4989**		Sheep	Abortion	Great Britain	nd	2	18	PCR	C	No
**LMNC150**	**JF4989**	**L101/2007**	Cattle	Rhombencephalitis	Switzerland	4b; 4d; 4e	1	1	HiSeq	A	No
**LMNC151**	**JF4981**		Sheep	Abortion	Great Britain	nd	2	20	PCR	C	No
**LMNC152**	**JF4979**		Sheep	Enteritis	Great Britain	nd	1	220	PCR	A	No
**LMNC153**	**JF4987**		Sheep	Enteritis	Great Britain	nd	1	220	PCR	A	No
**LMNC154**	**JF4986**		Sheep	Rhombencephalitis	Great Britain	nd	2	20	PCR	C	No
**LMNC155**	**JF4984**		Sheep	Abortion	Great Britain	nd	1	6	PCR	A	No
**LMNC156**	**JF4972**		Cattle	Abortion	Great Britain	nd	2	20	PCR	C	No
**LMNC157**	**JF5052***		Cattle	Mastitis	Switzerland	1/2a; 3a	2	412	HiSeq	C	Yes
**LMNC158**	**JF5519**		Cattle	Mastitis	Switzerland	4b; 4d; 4e	1	4	HiSeq	C	No
**LMNC159**	**JF5518**		Cattle	Mastitis	Switzerland	1/2a; 3a	2	29	HiSeq	C	No
**LMNC160**	**JF5448**		Sheep	Abortion	Switzerland	4b; 4d; 4e	1	219	HiSeq	A	No
**LMNC162**	**JF5529**		Sheep	Enteritis	Switzerland	nd	1	57	PCR	B	No
**LMNC163**	**JF5182***	**O/D36/08**	Cattle	Abortion	Switzerland	4b; 4d; 4e	1	1	HiSeq	A	Yes
**LMNC164**	**JF5336***	**A156**	Cattle	Abortion	Switzerland	1/2a; 3a	2	18	HiSeq	C	Yes
**LMNC165**	**JF5171***	**O/D1387/06**	Cattle	Abortion	Switzerland	1/2a; 3a	2	124	HiSeq	C	Yes
**LMNC166**	**JF5541**		Sheep	Rhombencephalitis	Switzerland	1/2a; 3a	2	403	HiSeq	nd	No
**LMNC167**	**JF5540**		Sheep	Rhombencephalitis	Switzerland	nd	1	4	PCR	nd	No

**LMNC168**	**JF5539**		Sheep	Rhombencephalitis	Switzerland	4b; 4d; 4e	1	4	HiSeq	nd	No
**LMNC169**	**JF5538**		Cattle	Rhombencephalitis	Switzerland	nd	1	1	PCR	nd	No
**LMNC170**	**JF5523**		Sheep	Enteritis	Switzerland	nd	2	16	PCR	C	No
**LMNC172**	**JF5809**		Goat	Floor	Switzerland	1/2c, 3c	2	37	HiSeq	nd	No
**LMNC173**	**JF5594**		Goat	Soil	Switzerland	4b; 4d; 4e	1	6	HiSeq	nd	No
**LMNC177**	**JF5810**		Goat	Soil	Switzerland	1/2a; 3a	2	26	HiSeq	nd	No
**LMNC234**	**JF5576**		Cattle	Mastitis	Switzerland	1/2a; 3a	2	412	PCR	Linked to C	No
**LMNC235**	**JF5577**		Goat	Abortion	Switzerland	nd	2	412	PCR	Linked to C	No
**LMNC236***	**JF5677**		Cattle	Water	Switzerland	1/2c, 3c	2	37	MiSeq	nd	Yes
**LMNC241***	**JF5679**		Cattle	Floor	Switzerland	1/2c, 3c	2	37	MiSeq	nd	Yes
**LMNC244***	**JF5680**		Cattle	Floor	Switzerland	1/2a; 3a	2	399	MiSeq	nd	Yes
**LMNC247***	**JF5801**		Cattle	Faeces	Switzerland	1/2a; 3a	2	399	MiSeq	nd	Yes
**LMNC250***	**JF5682**		Cattle	Feed bunk	Switzerland	1/2a; 3a	2	412	HiSeq	nd	Yes
**LMNC253***	**JF5683**		Cattle	Feed bunk	Switzerland	1/2a; 3a	2	399	MiSeq	nd	Yes
**LMNC257***	**JF5685**		Cattle	Water	Switzerland	1/2a; 3a	2	399	MiSeq	nd	Yes
**LMNC264***	**JF5687**		Cattle	Commodity feed	Switzerland	1/2c, 3c	2	37	MiSeq	nd	Yes
**LMNC265***	**JF5688**		Cattle	Soil	Switzerland	4b; 4d; 4e	1	4	MiSeq	nd	Yes
**LMNC266***	**JF5802**		Cattle	Soil	Switzerland	1/2c, 3c	2	37	MiSeq	nd	Yes
**LMNC269***	**JF5690**		Cattle	Soil	Switzerland	1/2a; 3a	2	7	MiSeq	nd	Yes
**LMNC270***	**JF5691**		Cattle	Floor	Switzerland	1/2c, 3c	2	29	MiSeq	nd	Yes
**LMNC271***	**JF5692**		Sheep	Rhombencephalitis	Switzerland	4b; 4d; 4e	1	4	MiSeq	nd	Yes
**LMNC272***	**JF5693**		Cattle	Rhombencephalitis	Switzerland	4b; 4d; 4e	1	1	MiSeq	nd	Yes
**LMNC273***	**JF5694**		Goat	Rhombencephalitis	Switzerland	1/2a; 3a	2	394	MiSeq	nd	Yes
**LMNC274***	**JF5695**		Cattle	Faeces	Switzerland	4b; 4d; 4e	1	4	MiSeq	nd	Yes
**LMNC277***	**JF5697**		Cattle	Feed bunk	Switzerland	4b; 4d; 4e	1	59	HiSeq	nd	Yes
**LMNC278***	**JF5698**		Cattle	Soil	Switzerland	4b; 4d; 4e	1	6	HiSeq	nd	Yes
**LMNC281***	**JF5699**		Cattle	Floor	Switzerland	4b; 4d; 4e	1	4	HiSeq	nd	Yes
**LMNC283***	**JF5701**		Cattle	Commodity feed	Italy	1/2a; 3a	2	36	MiSeq	nd	Yes
**LMNC284***	**JF5702**		Cattle	Soil	Italy	4b; 4d; 4e	1	663	MiSeq	nd	Yes
**LMNC287***	**JF5703**		Sheep	Faeces	Switzerland	1/2a; 3a	2	451	MiSeq	nd	Yes
**LMNC288***	**JF5704**		Sheep	Feed bunk	Switzerland	1/2a; 3a	2	451	MiSeq	nd	Yes
**LMNC290**	**JF5705**		Cattle	Abortion	Switzerland	1/2a	2	412	HiSeq	Linked to A	No
**LMNC291**	**JF5706**		Cattle	Abortion	Switzerland	1/2a	2	7	HiSeq	C	No
**LMNC292**	**JF5707**		Cattle	Abortion	Switzerland	1/2a	2	7	HiSeq	C	No
**LMNC293**	**JF5708**		Cattle	Abortion	Switzerland	1/2a	2	7	HiSeq	C	No
**LMNC294**	**JF5709**		Cattle	Abortion	Switzerland	1/2a	2	8	HiSeq	Linked to C	No
**LMNC295**	**JF5710**		Goat	Abortion	Switzerland	1/2a	2	29	HiSeq	Linked to C	No
**LMNC297***	**JF5711**		Sheep	Feed bunk	Switzerland	1/2a; 3a	2	394	MiSeq	nd	Yes
**LMNC300**	**JF5712**		Goat	Soil	Switzerland	4b; 4d; 4e	1	4	MiSeq	nd	No
**LMNC301***	**JF5713**		Goat	Soil	Switzerland	1/2a; 3a	2	18	MiSeq	nd	Yes
**LMNC302***	**JF5714**		Goat	Soil	Switzerland	4b; 4d; 4e	1	1	MiSeq	nd	Yes
**LMNC303***	**JF5715**		Goat	Floor	Switzerland	4b; 4d; 4e	1	4	MiSeq	nd	Yes
**LMNC307***	**JF5719**		Sheep	Soil	Switzerland	1/2a; 3a	2	11	MiSeq	nd	Yes
**LMNC314***	**JF5803**		Cattle	Feed bunk	Switzerland	1/2a; 3a	2	18	MiSeq	nd	Yes
**LMNC316***	**JF5722**		Cattle	Soil	Switzerland	4b; 4d; 4e	1	4	MiSeq	nd	Yes
**LMNC317***	**JF5723**		Cattle	Water	Switzerland	1/2c, 3c	2	37	MiSeq	nd	Yes
**LMNC318***	**JF5724**		Goat	Faeces	Germany	Lineage 3	3	70	MiSeq	nd	Yes
**LMNC319***	**JF5725**		Goat	Soil	Germany	1/2a; 3a	2	16	MiSeq	nd	Yes
**LMNC320***	**JF5726**		Goat	Soil	Germany	4b; 4d; 4e	1	1	MiSeq	nd	Yes
**LMNC323***	**JF5721**		Goat	Water	Germany	1/2a; 3a	2	200	MiSeq	nd	Yes
**LMNC325***	**JF5748**		Goat	Water	Germany	1/2a; 3a	2	204	MiSeq	nd	Yes
**LMNC326***	**JF5749**		Goat	Floor	Germany	Lineage 3	3	70	MiSeq	nd	Yes
**LMNC327***	**JF5750**		Goat	Floor	Germany	1/2a; 3a	2	16	MiSeq	nd	Yes
**LMNC328***	**JF5751**		Cattle	Commodity feed	Switzerland	4b; 4d; 4e	1	1	HiSeq	nd	Yes
**LMNC331***	**JF5754**		Cattle	Soil	Switzerland	4b; 4d; 4e	1	1	HiSeq	nd	Yes
**LMNC332***	**JF5755**		Goat	Feed bunk	Switzerland	1/2a; 3a	2	451	HiSeq	nd	Yes
**LMNC336***	**JF5759**		Goat	Soil	Switzerland	4b; 4d; 4e	1	4	HiSeq	nd	Yes
**LMNC337***	**JF5760**		Goat	Floor	Switzerland	4b; 4d; 4e	1	4	HiSeq	nd	Yes
**LMNC338***	**JF5761**		Sheep	Soil	Germany	1/2c, 3c	2	37	HiSeq	nd	Yes
**LMNC339***	**JF5762**		Sheep	Floor	Switzerland	1/2a; 3a	2	226	HiSeq	nd	Yes
**LMNC340***	**JF5763**		Cattle	Water	Switzerland	1/2a; 3a	2	399	HiSeq	nd	Yes
**LMNC351***	**JF5774**		Cattle	Water	Switzerland	1/2a; 3a	2	11	HiSeq	nd	Yes
**LMNC354***	**JF5777**		Cattle	Floor	Switzerland	1/2a; 3a	2	29	HiSeq	nd	Yes
**LMNC357***	**JF5780**		Cattle	Faeces	Switzerland	4b; 4d; 4e	1	1	HiSeq	nd	Yes
**LMNC358***	**JF5781**		Cattle	Commodity feed	Switzerland	4b; 4d; 4e	1	6	HiSeq	nd	Yes
**LMNC359***	**JF5782**		Cattle	Floor	Switzerland	1/2a; 3a	2	451	HiSeq	nd	Yes
**LMNC361***	**JF5784**		Cattle	Floor	Switzerland	1/2a; 3a	2	412	HiSeq	nd	Yes
**LMNC365***	**JF5788**		Cattle	Floor	Switzerland	1/2c, 3c	2	29	HiSeq	nd	Yes
**LMNC371***	**JF5794**		Cattle	Water	Switzerland	1/2a; 3a	2	226	HiSeq	nd	Yes
**LMNC378***			Goat	Rhombencephalitis	Switzerland	4b; 4d; 4e	1	1	HiSeq	nd	Yes
**LMNC379***			Goat	Rhombencephalitis	Switzerland	1/2c, 3c	2	37	HiSeq	nd	Yes
**LMNC380***			Goat	Rhombencephalitis	Switzerland	4b; 4d; 4e	1	1	HiSeq	nd	Yes
**LMNC381***			Goat	Rhombencephalitis	Switzerland	4b; 4d; 4e	1	1	HiSeq	nd	Yes
**LMNC382***			Sheep	Rhombencephalitis	Switzerland	4b; 4d; 4e	1	1	HiSeq	nd	Yes
**LMNC383***			Sheep	Rhombencephalitis	Switzerland	4b; 4d; 4e	1	4	HiSeq	nd	Yes
**LMNC384***			Sheep	Rhombencephalitis	Switzerland	4b; 4d; 4e	1	4	HiSeq	nd	Yes
**LMNC385**	**JF4907***	**L114/2007**	Cattle	Rhombencephalitis	Switzerland	4b; 4d; 4e	1	4	HiSeq	B	Yes
**LMNC387**	**JF5823**		Cattle	Abortion	Great Britain	1/2a;3a	2	91	HiSeq	nd	No
**LMNC388**	**JF5824**		Cattle	Abortion	Great Britain	4b; 4d; 4e	1	59	HiSeq	nd	No
**LMNC389**	**JF5825**		Cattle	Mastitis	Great Britain	1/2a;3a	2	20	HiSeq	nd	No
**LMNC391**	**JF5827**		Cattle	Abortion	Great Britain	1/2a;3a	2	7	HiSeq	nd	No
**LMNC392**	**JF5828**		Cattle	Abortion	Great Britain	1/2c, 3c	2	37	HiSeq	nd	No
**LMNC393**	**JF5829**		Cattle	Abortion	Great Britain	4b; 4d; 4e	1	59	HiSeq	nd	No
**LMNC394**	**JF5830**		Cattle	Abortion	Great Britain	1/2a;3a	2	18	HiSeq	nd	No

**LMNC395**	**JF5831**		Cattle	Abortion	Great Britain	1/2a;3a	2	7	HiSeq	nd	No
**LMNC396**	**JF5832**		Cattle	Abortion	Great Britain	1/2a;3a	2	177	HiSeq	nd	No
**LMNC397**	**JF5833**		Goat	Abortion	Great Britain	4b; 4d; 4e	1	220	HiSeq	nd	No
**LMNC409**			Sheep	Rhombencephalitis	Switzerland	4b; 4d; 4e	1	1	HiSeq	nd	No
**LMNC410**			Goat	Rhombencephalitis	Switzerland	1/2a; 3a	2	36	HiSeq	nd	No
**LMNC411**			Goat	Rhombencephalitis	Switzerland	4b; 4d; 4e	1	1	HiSeq	nd	No
**LMNC412**			Sheep	Rhombencephalitis	Switzerland	1/2c, 3c	2	91	HiSeq	nd	No
**LMNC413**			Sheep	Rhombencephalitis	Switzerland	4b; 4d; 4e	1	1	HiSeq	nd	No
**LMNC414**			Goat	Rhombencephalitis	Switzerland	4b; 4d; 4e	1	1	HiSeq	nd	No
**LMNC415**			Cattle	Rhombencephalitis	Switzerland	1/2a;3a	2	20	HiSeq	nd	No
**LMNC416**			Sheep	Rhombencephalitis	Switzerland	4b; 4d; 4e	1	1	HiSeq	nd	No
**LMNC417**			Cattle	Rhombencephalitis	Switzerland	1/2a;3a	2	412	HiSeq	nd	No
**LMNC418**			Cattle	Rhombencephalitis	Switzerland	4b; 4d; 4e	1	10	HiSeq	nd	No
**LMNC419**			Sheep	Rhombencephalitis	Switzerland	4b; 4d; 4e	1	1	HiSeq	nd	No
**LMNC420**			Cattle	Rhombencephalitis	Switzerland	4b; 4d; 4e	1	4	HiSeq	nd	No
**LMNC421**			Cattle	Rhombencephalitis	Switzerland	4b; 4d; 4e	1	1	HiSeq	nd	No
**LMNC422**			Cattle	Rhombencephalitis	Switzerland	1/2a;3a	2	412	HiSeq	nd	No
**LMNC423**			Cattle	Rhombencephalitis	Switzerland	4b; 4d; 4e	1	1	HiSeq	nd	No
**LMNC424**	**JF4903**	**L105/2007**	Cattle	Rhombencephalitis	Switzerland	4b; 4d; 4e	1	1	HiSeq	A	No
**LMNC425**	**JF4905**	**L110/2007**	Cattle	Rhombencephalitis	Switzerland	4b; 4d; 4e	1	1	HiSeq	A	No
**LMNC426**	**JF4910**	**L161/2008**	Cattle	Rhombencephalitis	Switzerland	1/2b; 3b; 7	1	59	HiSeq	B	No
**LMNC427**	**JF4911**	**L133/2007**	Cattle	Rhombencephalitis	Switzerland	4b; 4d; 4e	1	1	HiSeq	A	No
**LMNC428**	**JF4917**	**L11/2007**	Goat	Rhombencephalitis	Switzerland	4b; 4d; 4e	1	1	HiSeq	A	No
**LMNC429**	**JF4919**	**L32/2007**	Goat	Rhombencephalitis	Switzerland	4b; 4d; 4e	1	1	HiSeq	A	No
**LMNC430**	**JF4920**	**L40/2007**	Goat	Rhombencephalitis	Switzerland	1/2a; 3a	2	200	HiSeq	C	No
**LMNC431**	**JF4922**		Goat	Rhombencephalitis	Switzerland	1/2a;3a	2	20	HiSeq	nd	No
**LMNC432**	**JF4916**	**L9/2007**	Goat	Rhombencephalitis	Switzerland	4b; 4d; 4e	1	1	HiSeq	A	No
**LMNC433**	**JF4925**	**L95/2007**	Goat	Rhombencephalitis	Switzerland	1/2a; 3a	1	4	HiSeq	C	No
**LMNC434**	**JF4928**	**L130/2007**	Goat	Rhombencephalitis	Switzerland	4b; 4d; 4e	1	1	HiSeq	A	No
**LMNC435**	**JF4931**	**L51/2007**	Sheep	Rhombencephalitis	Switzerland	1/2a; 3a	2	415	HiSeq	C	No
**LMNC436**	**JF4932**	**L63/2007**	Sheep	Rhombencephalitis	Switzerland	4b; 4d; 4e	1	1	HiSeq	A	No
**LMNC437**	**JF4933**	**L83/2007**	Sheep	Rhombencephalitis	Switzerland	4b; 4d; 4e	1	1	HiSeq	A	No
**LMNC438**	**JF4938**	**L23/2007**	Sheep	Rhombencephalitis	Switzerland	1/2a; 3a	2	391	HiSeq	C	No
**LMNC439**	**JF4943**	**L39/2007**	Sheep	Rhombencephalitis	Switzerland	4b; 4d; 4e	1	1	HiSeq	A	No
**LMNC440**	**JF5143**	**L27/2007**	Sheep	Rhombencephalitis	Switzerland	4b; 4d; 4e	1	1	HiSeq	A	No
**LMNC441**	**JF5144**	**L28/2007**	Sheep	Rhombencephalitis	Switzerland	4b; 4d; 4e	1	1	HiSeq	A	No
**LMNC442**	**JF5145**	**L3/2007**	Sheep	Rhombencephalitis	Switzerland	4b; 4d; 4e	1	414	HiSeq	A	No
**LMNC443**	**JF5146**	**L33/2007**	Goat	Rhombencephalitis	Switzerland	4b; 4d; 4e	1	1	HiSeq	A	No
**LMNC444**	**JF5147**	**L34/2007**	Sheep	Rhombencephalitis	Switzerland	4b; 4d; 4e	1	1	HiSeq	A	No
**LMNC445**	**JF5148**	**L55/2007**	Sheep	Rhombencephalitis	Switzerland	4b; 4d; 4e	1	1	HiSeq	A	No
**LMNC446**	**JF5149**	**L56/2007**	Sheep	Rhombencephalitis	Switzerland	4b; 4d; 4e	1	1	HiSeq	A	No
**LMNC447**	**JF5151**	**L58/2007**	Sheep	Rhombencephalitis	Switzerland	4b; 4d; 4e	1	1	HiSeq	A	No
**LMNC448**	**JF5152**	**L59/2007**	Sheep	Rhombencephalitis	Switzerland	4b; 4d; 4e	1	1	HiSeq	A	No
**LMNC449**	**JF5153**	**L68/2007**	Sheep	Rhombencephalitis	Switzerland	4b; 4d; 4e	1	1	HiSeq	A	No
**LMNC450**	**JF5154**	**L69/2007**	Sheep	Rhombencephalitis	Switzerland	4b; 4d; 4e	1	1	HiSeq	A	No
**LMNC451**	**JF5155**	**L70/2007**	Sheep	Rhombencephalitis	Switzerland	4b; 4d; 4e	1	1	HiSeq	A	No
**LMNC452**	**JF5156**	**L71/2007**	Sheep	Rhombencephalitis	Switzerland	4b; 4d; 4e	1	1	HiSeq	A	No
**LMNC453**	**JF5158**	**L60/2007**	Sheep	Rhombencephalitis	Switzerland	4b; 4d; 4e	1	1	HiSeq	A	No
**LMNC454**	**JF5160**	**L64/2007**	Sheep	Rhombencephalitis	Switzerland	4b; 4d; 4e	1	1	HiSeq	A	No
**LMNC455**	**JF5161**	**L66/2007**	Sheep	Rhombencephalitis	Switzerland	4b; 4d; 4e	1	1	HiSeq	A	No
**LMNC456**	**JF5162**	**L67/2007**	Sheep	Rhombencephalitis	Switzerland	4b; 4d; 4e	1	1	HiSeq	A	No
**LMNC457**	**JF5163**	**L46/2007**	Goat	Rhombencephalitis	Switzerland	1/2a; 3a	2	26	HiSeq	Linked to C	No
**LMNC458**	**JF5165**	**L50/2007**	Goat	Rhombencephalitis	Switzerland	1/2a; 3a	2	415	HiSeq	Linked to C	No
**LMNC459**	**JF5168**	**L48/2007**	Goat	Rhombencephalitis	Switzerland	1/2a; 3a	2	412	HiSeq	C	No
**LMNC460**	**JF5169**	**L49/2007**	Goat	Rhombencephalitis	Switzerland	1/2a; 3a	2	7	HiSeq	C	No
**LMNC461**	**JF5170**	**L61/2007**	Sheep	Rhombencephalitis	Switzerland	1/2a; 3a	2	16	HiSeq	C	No
**LMNC462**	**JF5172***	**L10/2007**	Sheep	Rhombencephalitis	Switzerland	1/2a; 3a	2	16	HiSeq	Linked to C	Yes
**LMNC463**	**JF5179**	**L73/2007**	Sheep	Rhombencephalitis	Switzerland	4b; 4d; 4e	1	4	HiSeq	B	No
**LMNC464**	**JF5185**	**L4/2007**	Sheep	Rhombencephalitis	Switzerland	4b; 4d; 4e	1	414	HiSeq	Linked to A	No
**LMNC465**	**JF5192**	**L90/2007**	Sheep	Rhombencephalitis	Switzerland	4b; 4d; 4e	1	1	HiSeq	A	No
**LMNC466**	**JF5193**	**L92/2007**	Sheep	Rhombencephalitis	Switzerland	4b; 4d; 4e	1	1	HiSeq	A	No
**LMNC467**	**JF5198**	**L93/2007**	Sheep	Rhombencephalitis	Switzerland	4b; 4d; 4e	1	1	HiSeq	A	No
**LMNC468**	**JF5200**	**L140/2007**	Cattle	Rhombencephalitis	Switzerland	4b; 4d; 4e	1	1	HiSeq	A	No
**LMNC469**	**JF5204**	**L88/2007**	Sheep	Rhombencephalitis	Switzerland	4b; 4d; 4e	1	1	HiSeq	A	No
**LMNC470**	**JF5205**	**L85/2007**	Sheep	Rhombencephalitis	Switzerland	4b; 4d; 4e	1	1	HiSeq	A	No
**LMNC471**	**JF5206**	**L147/2008**	Sheep	Rhombencephalitis	Switzerland	4b; 4d; 4e	1	1	HiSeq	A	No
**LMNC472**	**JF5209**	**L123/2007**	Cattle	Rhombencephalitis	Switzerland	4b; 4d; 4e	1	1	HiSeq	A	No
**LMNC474**	**JF5176**	**L74/2007**	Sheep	Rhombencephalitis	Switzerland	1/2a; 3a	2	21	HiSeq	Linked to C	No
**LMNC475**	**JF5177**	**L76/2007**	Sheep	Rhombencephalitis	Switzerland	1/2a; 3a	2	591	HiSeq	Linked to C	No
**LMNC477**	**JF5211**	**O/D115/04**	Sheep	Rhombencephalitis	Switzerland	1/2a; 3a	2	412	PCR	C	No
**LMNC478**	**JF4899**		Cattle	Rhombencephalitis	Switzerland	4b; 4d; 4e	1	1	HiSeq	nd	No
**LMNC479**	**JF4901***	**L103/2007**	Cattle	Rhombencephalitis	Switzerland	4b; 4d; 4e	1	1	HiSeq	A	Yes
**LMNC480**	**JF4902***	**L104/2007**	Cattle	Rhombencephalitis	Switzerland	4b; 4d; 4e	1	1	HiSeq	A	Yes
**LMNC481**	**JF4906***	**L113/2007**	Cattle	Rhombencephalitis	Switzerland	4b; 4d; 4e	1	413	HiSeq	A	Yes
**LMNC482**	**JF4909***	**L117/2007**	Cattle	Rhombencephalitis	Switzerland	4b; 4d; 4e	1	1	HiSeq	A	Yes
**LMNC483**	**JF4927***	**L129/2007**	Goat	Rhombencephalitis	Switzerland	4b; 4d; 4e	1	1	HiSeq	A	Yes
**LMNC484**	**JF5159***	**L62/2007**	Sheep	Rhombencephalitis	Switzerland	4b; 4d; 4e	1	1	HiSeq	A	Yes
**LMNC485**	**JF5181***	**L82/2007**	Sheep	Rhombencephalitis	Switzerland	4b; 4d; 4e	1	1	HiSeq	A	Yes
**LMNC486**	**JF5183***	**O/D562/09**	Cattle	Rhombencephalitis	Switzerland	4b; 4d; 4e	1	1	HiSeq	A	Yes
**LMNC487**	**JF5199***	**L118/2007**	Cattle	Rhombencephalitis	Switzerland	4b; 4d; 4e	1	1	HiSeq	A	Yes
**LMNC488**	**JF5207***	**L119/2007**	Cattle	Rhombencephalitis	Switzerland	4b; 4d; 4e	1	515	HiSeq	A	Yes
**LMNC489**	**JF5210***	**L134/2007**	Cattle	Rhombencephalitis	Switzerland	4b; 4d; 4e	1	1	HiSeq	A	Yes
**LMNC491**	**JF5404**		Goat	Mastitis	Switzerland	nd	2	8	PCR	Linked to C	No
**LMNC542**			Sheep	Rhombencephalitis	Switzerland	4b; 4d; 4e	1	4	HiSeq	nd	No

**JF4980**		Sheep	Enteritis	Great Britain	nd	1	5	PCR	Linked to A	No
**JF5202**	**L145/2007**	Cattle	Rhombencephalitis	Switzerland	4b; 4d; 4e	1	1	HiSeq	A	No

***: ID used in this study for virulence analysis**
nd: non detrmined

Table. S2

Summary of virulence-associated gene analysis

Virulence protein	Virulence gene	Nucleotide alignment			Amino acid alignment			Recombination analysis			Known point mutations[e]	Missing in STs
		Allele No.	Lineage separation	Divergence	Allele No.	Lineage separation	Divergence	No. of recombinations	r/m[c]	ρ/θ[d]		
ActA	*actA*	26	Yes	According to ST	21	Yes	Shared alleles[a]	22	3.92 (3.37-4.47)	0.57 (0.50-0.64)		
CtaP	*ctaP*	29	Yes	According to ST	8	No	Shared alleles[a]	15	8.11 (5.21-11.02)	2.63 (1.78-3.48)		
DltA	*dltA*	12	Yes	Shared alleles[b]	17	Yes	Shared alleles[b]	4	4.87 (4.48-5.26)	0.28 (0.24-0.33)		
FbpA	*fbpA*	12	Yes	Shared alleles[a]	7	Yes	Shared alleles[a]	3	5.82 (5.20-6.43)	0.17 (0.14-0.20)		
Fri	*fri*	6	Yes	Shared alleles[a]	8	No	Shared alleles[a]	2	0.78 (0.55-1.00)	0.13 (0.06-0.21)		
GAPDH	*gap*	10	Yes	Shared alleles[a]	10	No	Shared alleles[a]	1	2.15 (-3.35-7.67)	2.1 (-4.47-8.69)		
GtcA	*gtcA*	7	Yes	Shared alleles[a]	6	Yes	Shared alleles[a]	2	41.92 (12.62-71.22)	2.72 (0.44-4.99)		
Hfq	*hfq*	3	Yes	According to lineage	1	No	None	2	0.33 (-0.38-1.047)	0.42 (0.22-0.62)		
Hpt	*uhpT*	23	Yes	According to ST	12	Yes	Shared alleles[a]	6	1.61 (1.53-1.69)	0.26 (0.23-0.28)		
InlA	*inlA*	23	Yes; except Lineage III	According to ST	19	Yes; except Lineage III	Shared alleles[a]	19	16.8 (14.24-19.42)	3.91 (3.26-4.57)		
InlB	*inlB*	23	Yes	According to ST	22	Yes	According to ST	10	3.52 (2.31-4.74)	0.5 (0.42-0.58)		
InlC	*inlC*	15	Yes	Shared alleles[a]	14	Yes	Shared alleles[a]	5	1.46 (-0.32-3.25)	0.32 (-0.31-0.96)		
InlF	*inlF*	23	Yes; except Lineage III	According to ST	21	Yes; except Lineage III	Shared alleles[a]	4	7.41 (7.18-7.64)	0.16 (0.12-0.20)		ST11; 70; 399
InlH	*inlH*	22	Yes	According to ST	17	Yes	Shared alleles[a]	18	7.68 (7.08-8.28)	1.09 (1.00-1.17)		
InlJ	*inlJ*	25	Yes	According to ST	25	Yes	According to ST	24	6.39 (6.06-6.71)	1.17 (0.99-1.35)		
Lap	*lap*	20	Yes	Shared alleles[a]	4	Yes	Shared alleles[a]	5	8.46 (7.20-9.71)	1.01 (0.81-1.22)		
LapB	*lapB*	32	Yes; except Lineage III	According to ST	23	Yes; except Lineage III	According to ST	19	4.52 (4.37-4.66)	0.34 (0.31-0.38)		ST70; 412
Lgt	*lgt*	8	Yes	Shared alleles[a]	4	Yes	Shared alleles[a]	3	3.17 (1.18-5.15)	0.28 (0.09-0.46)		
LLO	*hly*	20	Yes	Shared alleles[a]	10	Yes	Shared alleles[a]	4	0.49 (0.18-0.79)	0.03 (0.007-0.05)		
LntA	*lntA*	17	Yes	Shared alleles[a]	17	Yes	Shared alleles[a]	16	6.89 (6.46-7.32)	1.03 (0.94-1.12)		
LpeA	*lpeA*	13	Yes	Shared alleles[a]	12	Yes	Shared alleles[a]	4	2.93 (2.67-3.18)	0.18 (0.15-0.20)		
LplA1	*lplA1*	22	Yes	According to ST	12	Yes	Shared alleles[a]	13	2.2 (0.06-4.33)	0.17 (-0.25-0.61)		
Lsp	*lsp*	5	Yes	Shared alleles[a]	3	Yes	Shared alleles[a]	3	3.67 (-0.77-8.11)	0.68 (-0.11-1.48)		
Mpl	*mpl*	18	Yes	Shared alleles[a]	12	Yes	Shared alleles[a]	5	2.03 (-0.17-4.23)	0.17 (-0.03-0.37)		
MprF	*mprF*	18	Yes	Shared alleles[a]	9	Yes	Shared alleles[a]	3	0.57 (-0.18-1.33)	0.01 (-0.006-0.03)		
MurA	*murA*	21	Yes	According to ST	19	Yes	Shared alleles[a]	14	4.2 (4.04-4.37)	0.52 (0.49-0.55)		
OppA	*oppA*	13	Yes	Shared alleles[a]	7	Yes	Shared alleles[a]	2	0.58 (0.29-0.86)	0.06 (-0.04-0.17)		
P60	*iap*	25	Yes	According to ST	14	Yes	Shared alleles[a]	16	3.88 (3.31-4.44)	0.02 (0.01-0.04)		
PgdA	*pgdA*	12	Yes	Shared alleles[b]	9	Yes	Shared alleles[b]	4	0.63 (0.32-0.94)	0.03 (-0.04-0.11)		
Pgl	*pgl*	12	Yes	Shared alleles[a]	16	Yes	Shared alleles[a]	7	1.37 (1.17-1.56)	0.18 (0.14-0.22)		
PlcA	*plcA*	18	Yes	Shared alleles[a]	13	Yes	Shared alleles[a]	8	2.01 (1.67-2.36)	0.41 (0.26-0.55)	**I17V**: ST394; 412 **F119Y**: ST11; 16;18;36;37;124;394;399;412;451	
PlcB	*plcB*	17	Yes	Shared alleles[a]	18	Yes	Shared alleles[a]	3	0.5 (0.28-0.72)	0.03 (0.01-0.05)	**A223V**: ST70 **D61E**: ST1: 413; 515	
PrfA	*prfA*	12	Yes	Shared alleles[a]	9	Yes	Shared alleles[a]	3	0.3 (-0.11-0.72)	0.04 (-0.07-0.16)		
PrsA2	*prsA2*	9	Yes	Shared alleles[a]	7	Yes	Shared alleles[a]	3	0.52 (-0.07-1.12)	0.05 (-0.0005-0.11)		
PycA	*pycA*	24	Yes	According to ST	11	Yes	Shared alleles[a]	8	4.64 (4.41-4.87)	0.31 (0.29-0.32)		
RecA	*recA*	5	Yes	Shared alleles[a]	5	Yes; except Lineage III	Shared alleles[a]	2	13.79 (10.71-16.87)	0.91 (0.69-1.13)		
RelA	*relA*	16	Yes	Shared alleles[a]	6	Yes	Shared alleles[a]	3	2.63 (2.30-2.96)	0.09 (0.07-0.11)		
SecA2	*secA2*	18	Yes	Shared alleles[a]	9	Yes	Shared alleles[a]	5	2.7 (2.34-3.06)	0.11 (0.09-0.13)		
SigB	*sigB*	7	Yes	Shared alleles[a]	5	Yes; except Lineage III	Shared alleles[a]	1	1.92 (1.45-2.39)	0.29 (0.20-0.39)		
SipZ	*sipZ*	8	Yes	Shared alleles[a]	7	Yes	Shared alleles[a]	2	1.06 (-0.51-2.63)	0.1 (-0.06-0.26)		
Sod	*sod*	10	Yes	Shared alleles[a]	7	Yes	Shared alleles[a]	4	0.7 (-0.37-1.78)	0.18 (-0.13-0.50)		
SrtA	*srtA*	11	Yes	Shared alleles[a]	6	Yes	Shared alleles[a]	5	4.47 (4.23-4.72)	0.7 (0.66-0.74)		
SvpA	*svpA*	14	Yes	Shared alleles[a]	11	Yes	Shared alleles[a]	3	3.11 (2.68-3.55)	0.16 (0.14-0.19)		
Vip	*vip*	17	Yes; except Lineage III	Shared alleles[a]	15	Yes; except Lineage III	Shared alleles[a]	9	4.08 (3.14-5.02)	0.02 (0.01-0.03)		ST16; 29; 37; 70; 226; 412
VirR	*virR*	10	Yes	Shared alleles[a]	4	No; just Lineage III	Shared alleles[a]	2	2.1 (1.61-2.58)	0.13 (0.08-0.19)		

a: alleles shared between STs of the same lineage
b: alleles shared between STs of different lineages
c: relative impact of recombination compared to point mutation, mean values and 95 % CI of 5 individual runs
d: relative frequency of recombination occurrence compared to point mutation , mean values and 95 % CI of 5 individual runs
e: Roche et al., 2005

Table. S3

Summary of the sampled farms

Farm no.	Date	Country	Species Type	Herd size (no. of animals)	Listeriosis cases	No. of positive samples	Positive samples
**1**	17.02.2014	Switzerland	Goats	60	yes	0	none
**2**	18.02.2014	Switzerland	Sheep	230	yes	2	soil / water
**3**	24.02.2014	Switzerland	Cattle	22	yes	0	none
**4**	05.05.2014	Switzerland	Cattle	40	no	1	silage
**5**	12.05.2014	Switzerland	Cattle	40	no	0	none
**6**	19.05.2014	Switzerland	Cattle	40	yes	3	water / floor / feed bunk
**7**	26.05.2014	Switzerland	Cattle	43	yes	0	none
**8**	02.06.2014	Switzerland	Goats	25	yes	2	soil / floor
**9**	20.10.2014	Switzerland	Cattle	50	no	4	faeces / feed bunk / water / floor
**10**	03.11.2014	Switzerland	Cattle	80	no	3	commodity feed / soil / floor
**11**	24.11.2014	Switzerland	Cattle	100	yes	4	faeces / feed bunk / soil / floor
**12**	29.11.2014	Italy	Cattle	160	no	2	commodity feed / soil
**13**	01.12.2014	Switzerland	Sheep	40	no	2	faeces / feed bunk
**14**	08.12.2014	Switzerland	Sheep	24	yes	0	none
**15**	05.01.2015	Switzerland	Sheep	100	yes	1	feed bunk
**16**	19.01.2015	Switzerland	Sheep	200	yes	1	soil
**17**	26.01.2015	Switzerland	Sheep	25	yes	0	none
**18**	02.02.2015	Switzerland	Goats	77	yes	0	none
**19**	09.02.2015	Switzerland	Cattle	70	yes	3	feed bunk / soil / water
**20**	04.02.2015	Germany	Goats	360	yes	4	faeces / soil / water / floor
**21**	16.02.2014	Switzerland	Sheep	80	no	0	none
**22**	02.03.2015	Switzerland	Cattle	20	yes	2	commodity feed / soil
**23**	09.03.2015	Switzerland	Goats	20	yes	3	feed bunk / soil / floor
**24**	11.03.2015	Germany	Sheep	1600	yes	0	none
**25**	13.03.2015	Germany	Sheep	220	yes	1	soil
**26**	23.03.2015	Switzerland	Sheep	80	yes	1	floor
**27**	24.03.2015	Germany	Goats	44	no	0	none
**28**	07.04.2015	Switzerland	Cattle	42	no	1	water
**29**	07.04.2015	Switzerland	Cattle	20	no	1	floor
**30**	07.04.2015	Switzerland	Cattle	40	no	3	faeces / commodity feed / floor
**31**	10.04.2015	Switzerland	Cattle	30	no	3	soil / water / floor
**32**	10.04.2015	Switzerland	Cattle	80	no	0	none

Table. S4

L. monocytogenes detection in 224 samples from 32 ruminant farms

Samples	No. of samples positive for *L. monocytogenes*				
	**All farms (n=32) (%)**	**Small ruminant farms (n=16) (%)**	**Cattle farms (n=16) (%)**	**Control farms (n=13) (%)**	**Case farms (n=19) (%)**
**Faeces**	5 (16)	2 (12)	3 (19)	3 (23)	2 (10)
**Soil**	12 (37)	6 (37)	6 (37)	3 (23)	9 (47)
**Silage**	1 (3)	0 (0)	1 (6)	1 (8)	0 (0)
**Commodity feeds**	4 (12)	0 (0)	4 (25)	3 (23)	1 (5)
**Feed bunk swabs**	7 (22)	3 (19)	4 (25)	2 (12)	5 (26)
**Water tank swabs**	7 (22)	2 (12)	5 (31)	3 (23)	4 (21)
**Floor swabs**	11 (34)	4 (25)	7 (44)	5 (38)	6 (31)
**TOTAL**	47 (21)	17 (15)	30 (27)	20 (22)	27 (20)
